# Supplementary material for: Complex behavior from intrinsic motivation to occupy future action-state path space
Source: Nat Commun. 2024 Jul 29;15:6368. doi: 10.1038/s41467-024-49711-1 (PMC11286966; doi:10.1038/s41467-024-49711-1)
Supplement: Supplementary file 1 — Supplementary Information [file 41467_2024_49711_MOESM1_ESM.pdf]

Supplemental material for "Complex behavior from intrinsic motivation to occupy action-state path space"

A Entropy measures the occupancy of action-state paths

In this section, we show that entropy is the only measure of action-state path occupancy that obeys some basic intuitive notions of occupancy. We first list the intuitive conditions in mathematical form, present the main theorem and then discuss some implications through some corollaries.

We consider a time-homogeneous Markov decision process with finite state set $\mathcal{S}$ and finite action set $\mathcal{A}(s)$ for every state $s \in \mathcal{S}$. Henceforth, the action-state $x_j = (a_j, s_j)$ is any joint pair of one available action a_j and one possible successor state s_j that results from making that action under policy $\pi \equiv \{\pi(a|s)\}$ from the action-state $x_i = (a_i, s_i)$. By assumption, the availability of action a_j depends on the previous state s_i alone, not on a_i. Thus, the transition probability from x_i to x_j in one time step is $p_{ij} = \pi(a_j|s_i)p(s_j|s_i, a_j)$, where $p(s_j|s_i, a_i)$ is the conditional probability of transitioning from state s_i to s_j given that action a_j is performed. Although there is no dependence of the previous action a_i on this transition probability, it is notationally convenient to define transitions between action-states. We conceive of rational agents as maximizing future action-state path occupancy. Any measure of occupancy should obey the intuitive Conditions *1-4* listed below.

Intuitive Conditions for a measure of action-state occupancy:

1. *Occupancy gain of action-state x_j from x_i is a function of the transition probability p_{ij}, $C(p_{ij})$*

2. *Performing a low probability transition leads to a higher occupancy gain than performing a high probability transition, that is, $C(p_{ij})$ decreases with p_{ij}*

3. *The first order derivative $C'(p_{ij})$ is continuous for $p_{ij} \in (0, 1)$*

4. *(Definition: the action-state occupancy of a one-step path from action-state x_i is the expectation over occupancy gains of the immediate successor action-states, $C_i^{(1)} \equiv \sum_j p_{ij}C(p_{ij})$)*

   The action-state occupancy of a two-steps path is additive,

   $C_i^{(2)} \equiv \sum_{jk} p_{ij}p_{jk}C(p_{ij}p_{jk}) = C_i^{(1)} + \sum_j p_{ij}C_j^{(1)}$

   for any choice of the p_{ij} and initial x_i

Condition *1* simply states that occupancy gain from an initial action-state is defined over the transition probabilities to successor action-states in a sample space. Condition *2* implies that performing a low probability transition leads to a higher occupancy of the successor states than performing a high probability transition. This is because performing a rare transition allows the agent to occupy a space that was left initially unoccupied. Condition *3* imposes smoothness of the measure.

In Condition *4* we have defined the occupancy of the successor action-states (one-step paths) in the Markov chain as the expected occupancy gain. Condition *4* is the central property, and it imposes that the occupancy of action-states paths with two steps can be broken down into a sum of the occupancies of action-states at each time step. Note that the action-state path occupancy can be written as

$$C_i^{(2)} \equiv \sum_{jk} p_{ij}p_{jk}C(p_{ij}p_{jk}) = \sum_j p_{ij}C(p_{ij}) + \sum_{jk} p_{ij}p_{jk}C(p_{jk}) = \sum_{jk} p_{ij}p_{jk}\left(C(p_{ij}) + C(p_{jk})\right),$$

which imposes a strong condition on the function $C(p)$. Note also that the sum $\sum_{jk} p_{ij}p_{jk}C(p_{ij}p_{jk})$ extends the notion of action-state to a path of two consecutive action-states, each path having probability $p_{ij}p_{jk}$ due to the (time-homogeneous) Markov property. The last equality is an identity. While here we consider paths of length equal to 2, further below we show that there is no difference in imposing additivity to paths of any fixed or random length (Corollary 2).

Theorem 1. *$C(p) = -k \ln p$ with $k > 0$ is the only function that satisfies Conditions* 1-4

Corollary 1. *The entropy $C_i^{(1)} = -k \sum_j p_{ij} \ln p_{ij}$ is the only measure of action-state occupancy of successor action-states x_j from x_i with transition probabilities p_{ij} consistent with Conditions* 1-4.

Proof. Put $p_{1,1} = 1$ and $p_{1,j} = 0$ for $j \neq 1$. Then, Condition *4* reads $C(1) = C(1) + C(1)$ when the initial action-state is x_1, which implies $C(1) = 0$.

Now, take a Markov chain with $p_{0,0} = 1$, $p_{1,0} = 1 - t > 0$, $p_{1,2} = t > 0$, $p_{2,0} = p_{2,1} = 0$, $p_{2,j} = 1/n$ for $j = 3, ..., n + 2$ and $n > 0$, and $p_{k,0} = 1$ for $k = 3, ..., n + 2$. In this chain, the state 0 is absorbing and all others are transient (here action-states are simply referred to as states). Starting from state 1, transition to the transient state 2 happens with probability t and to the absorbing state 0 with probability $1 - t$. From state 2 a transition to states $j = 3, ..., n + 2$ happens with equal probability. From any of those states, a deterministic transition to 0 ensues. (These last transitions can only happen in the third time step, and although it will be relevant later on, it is no used in the current proof, which only uses additivity on paths of length two.) Then, Condition *4* with initial state 1 reads $tC(t/n) + (1 - t)C(1 - t) = tC(t) + (1 - t)C(1 - t) + tC(1/n) + (1 - t)C(1)$, and hence $C(t/n) = C(t) + C(1/n)$ for any $0 < t < 1$ and integer $n > 0$. By Condition *3* and taking derivative with respect to t in both sides, we obtain $C'(t/n) = nC'(t)$, and multiplying in both sides by t we obtain $\frac{t}{n}C'(\frac{t}{n}) = tC'(t)$. By replacing t with nt, we get $tC'(t) = ntC'(nt)$, provided that $nt < 1$.

We will now show that $tC'(t)$ is constant. In the last equation replace t by t/m by integer $m > 0$ to get the last equivalence in $tC'(t) = \frac{t}{m}C'(\frac{t}{m}) = \frac{n}{m}tC'(\frac{n}{m}t)$ (the first equivalence is obvious). These equivalences are valid for positive $t < 1$ and $\frac{n}{m}t < 1$. Let $0 < s < 1$ and $n = \lfloor ms/t \rfloor$ be the largest integer smaller than ms/t. Therefore, as m increases $\frac{n}{m}t < 1$ and approaches s as close as desired. By Condition *3* the function $xC'(x)$ is continuous, and therefore $\lim_{m\to\infty} \frac{n}{m}tC'(\frac{n}{m}t) = sC'(s)$. The basic idea is that we can first compress t as much as needed by the integer factor m and then expand it by the integer factor n so that nt/m is as close as desired to s. This shows that $sC'(s) = tC'(t)$ for $s, t \in (0, 1)$, and therefore $tC'(t)$ is constant.

Assume that $tC'(t) = -k$. Then, by integrating we obtain $C(t) = -k \ln t + a$, but $a = 0$ due to $C(1) = 0$, and $k > 0$ due to Condition *2*. Together with the above, we can now proof the theorem by noticing that the solution satisfies Condition *4* for any choice of the p_{ij}. □

Remark: We have found that entropy is the measure of occupancy. The famous derivation of entropy as a measure of information [78] uses similar elements, but some differences are worthy to be mentioned. First, our proof uses the notion of additivity of occupancy on MDPs of length two (our Condition *4*), while Shannon's notion of additivity uses sequences of random variable of arbitrary length (his Condition *3*), and therefore his condition is in a sense stronger than ours. Second, our proof enforces continuous derivative of the measure, while Shannon enforces continuity of the measure, rendering our Condition *3* stronger. Finally, we enforce a specific form of the measure as an average over occupancy gains (our Condition *4* again), because it intuitively captures the notion of occupancy, while Shannon does not enforce this structure in his information measure.

Corollary 2. Condition *4* can be replaced by the stronger condition that requires additivity of paths of any finite length n with no change in the above proof. We first introduce some notation: the probability

of path $i_0, i_1, ..., i_n$ is $p_{i_0,i_1}p_{i_1,i_2}...p_{i_{n-1},i_n}$, where i_t refers to the state visited at step t and i_0 is the initial state. Then the new Condition *4* reads in terms of the action-state occupancy of paths of length n as

$$
\begin{aligned}
C_{i_0}^{(n)} &= \sum_{i_1,i_2,...,i_n} p_{i_0,i_1}p_{i_1,i_2}...p_{i_{n-1},i_n} C\left(p_{i_0,i_1}p_{i_1,i_2}...p_{i_{n-1},i_n}\right) \\
&= \sum_{i_1} p_{i_0,i_1}C(p_{i_0,i_1}) + \sum_{i_1,i_2} p_{i_0,i_1}p_{i_1,i_2}C(p_{i_1,i_2}) + ... + \sum_{i_1,i_2,...,i_n} p_{i_0,i_1}p_{i_1,i_2}...p_{i_{n-1},i_n} C\left(p_{i_{n-1},i_n}\right) \\
&= \sum_{i_1,i_2,...,i_n} p_{i_0,i_1}p_{i_1,i_2}...p_{i_{n-1},i_n}\left(C(p_{i_0,i_1}) + C(p_{i_1,i_2})... + C(p_{i_{n-1},i_n})\right) ,
\end{aligned}
$$

for any time-homogeneous Markov chain. By choosing the particular chains used in Theorem 1, we arrive again to the same unique solution $C(p) = -k \ln p$ after using $C(1) = 0$ repeated times, which obviously solves the above equation for any chain and length path. Indeed, note that for the second chain in Theorem 1, from initial state 1 the absorbing state is reached in three time steps with probability one, and thus the above sum contains all $C(1)$ starting from the third terms, which contribute zero to the sum.

The above entropy measure of action-state path occupancy can be extended to the case where there is a discount factor $0 < \gamma < 1$. To do so, we assume now that the paths can have a random length $n \geq 1$ that follows a geometric distribution, $p_n = \gamma^{n-1}(1-\gamma)$. In this case, the occupancy of the paths is

$$
\begin{aligned}
C_{\text{global}} &= (1-\gamma)\sum_{i_1} p_{i_0,i_1}C(p_{i_0,i_1}) + \gamma(1-\gamma)\sum_{i_1,i_2} p_{i_0,i_1}p_{i_1,i_2}C(p_{i_0,i_1}p_{i_1,i_2}) \\
&\quad +\gamma^2(1-\gamma)\sum_{i_1,i_2,i_3} p_{i_0,i_1}p_{i_1,i_2}p_{i_2,i_3}C(p_{i_0,i_1}p_{i_1,i_2}p_{i_2,i_3}) + ... \qquad \text{(A.1)}
\end{aligned}
$$

where the n-th term in the sum is the expected occupancy gain of paths of length n weighted by the probability of a having a path with exactly such a length.

Equivalently, a path in course can grow one step further with probability γ or be extinguished with probability $1-\gamma$. Therefore, the occupancy in Eq. (A.1) should also be equal to the sum of the expected occupancy gains of the local states along the paths, defined as

$$
C_{\text{local}} = \sum_{i_1} p_{i_0,i_1}C(p_{i_0,i_1}) + \gamma\sum_{i_1,i_2} p_{i_0,i_1}p_{i_1,i_2}C(p_{i_1,i_2}) + \gamma^2\sum_{i_1,i_2,i_3} p_{i_0,i_1}p_{i_1,i_2}p_{i_2,i_3}C(p_{i_2,i_3}) + ... \qquad \text{(A.2)}
$$

where the first term is the expected occupancy gain given by the initial condition, the second term is the expected occupancy gain in the next step weighted by the probability of having a path length of at least two steps, and so on.

Eqs. (A.1-A.2), after using the Markov chain in Corollary 2, reduce to

$$
\begin{aligned}
C_{\text{global}} &= (1-\gamma)\sum_{i_1} p_{i_0,i_1}C(p_{i_0,i_1}) + \gamma(1-\gamma)\sum_{i_1,i_2} p_{i_0,i_1}p_{i_1,i_2}C(p_{i_0,i_1}p_{i_1,i_2}) \\
&\quad +\gamma^2(1-\gamma)\sum_{i_1,i_2} p_{i_0,i_1}p_{i_1,i_2}C(p_{i_0,i_1}p_{i_1,i_2}) + ... \\
&= (1-\gamma)\sum_{i_1} p_{i_0,i_1}C(p_{i_0,i_1}) + \gamma\sum_{i_1,i_2} p_{i_0,i_1}p_{i_1,i_2}C(p_{i_0,i_1}p_{i_1,i_2})
\end{aligned}
$$

and

$$
C_{\text{local}} = \sum_{i_1} p_{i_0,i_1}C(p_{i_0,i_1}) + \gamma\sum_{i_1,i_2} p_{i_0,i_1}p_{i_1,i_2}C(p_{i_1,i_2}),
$$

where we have used $p_{i_2,i_3} = 1$ because all transitions in the third step are deterministic.

Equality of these two quantities leads to Condition *4*, specifically, $\sum_{i_1,i_2} p_{i_0,i_1}p_{i_1,i_2}C(p_{i_0,i_1}p_{i_1,i_2}) = \sum_{i_1} p_{i_0,i_1}C(p_{i_0,i_1}) + \sum_{i_1,i_2} p_{i_0,i_1}p_{i_1,i_2}C(p_{i_1,i_2})$. Therefore, the only consistent measure of occupancy with temporal discount is the entropy. Obviously, the equality of global and local time-discounted occupancies measured by entropy holds for any time-homogeneous or inhomogeneous Markov chain.

B Critical policies and critical state-value functions

Here, the expected return following policy π in Eq. (A.2), known as the state-value function, is written recursively using the Bellman equation. Then, we find a non-linear system of equations for the critical policy and critical state-value function by taking partial derivatives with respect to the policy probabilities (Theorem 2).

Using Eq. (A.2) and Theorem 1 with $k = 1$, we define the expected return from state s under policy π as

$$V_\pi(s) = -\sum_{i_1} p_{s,i_1} \ln p_{s,i_1} - \gamma \sum_{i_1,i_2} p_{s,i_1}p_{i_1,i_2} \ln p_{i_1,i_2} - \gamma^2 \sum_{i_1,i_2,i_3} p_{s,i_1}p_{i_1,i_2}p_{i_2,i_3} \ln p_{i_2,i_3} + ... \quad \text{(B.1)}$$

where p_{s,i_1} is the transition probability from state s to action-state $x_{i_1} = (a_{i_1}, s_{i_1})$. Note that in Eq. (A.2) we have replaced the initial action-state i_0 by the initial state s alone, as the previous action that led to it does no affect the transition probabilities in the Markov decision process setting. The expected returns satisfy the standard recurrence relationship [14]

$$\begin{aligned} V_\pi(s) &= \sum_{a,s'} p_{s,(a,s')} \left(-\ln p_{s,(a,s')} + \gamma V_\pi(s')\right) \\ &= \sum_{a,s'} \pi(a|s)p(s'|s,a) \left(-\ln \pi(a|s)p(s'|s,a) + \gamma V_\pi(s')\right). \end{aligned} \quad \text{(B.2)}$$

Here, we have unpacked the sum over the action-state i_1 into a sum over (a, s'), where a is the action made in state s and s' is its successor. The second equation shows, in a more standard notation, the explicit dependence of the expected return on the policy. It also highlights that the intrinsic immediate reward takes the form $R_{\text{intrinsic}}(s, a, s') = -\ln \pi(a|s)p(s'|s, a)$, which is unbounded.

From Eq. (B.1) it is easy to see that the expected return exists (is finite) for any policy π if the Markov decision process has a finite number of actions and states. Due to the properties of entropy, Eq. (B.1) is a sum of non-negative numbers bounded by $H_{max} = \ln(|A|_{max}|S|)$ ($|A|_{max}$ is the maximum number of available actions from any state) weighted by the geometric series, which guarantees convergence of the infinite sum for $-1 < \gamma < 1$. An obvious, but relevant, implication of the above is that the expected return is non-negative and bounded, $0 \le V_\pi(s) \le H_{max}/(1 - \gamma)$, for any state and policy.

While in Eq. (B.2) the immediate intrinsic reward is the sum of the action and state occupancies, $R_{\text{intrinsic}}(s, a, s') = -\ln \pi(a|s)p(s'|s, a) = -\ln \pi(a|s) - \ln p(s'|s, a)$, we can generalize this reward to consider any weighted mixture of entropies as $R_{\text{intrinsic}}(s, a, s') = -\alpha \ln \pi(a|s) - \beta \ln p(s'|s, a)$ for any two numbers $\alpha > 0$ and $\beta \ge 0$. In particular, for $(\alpha, \beta) = (1, 1)$ we recover the action-state occupancy of Eq. (B.2), and for $(\alpha, \beta) = (1, 0)$ and $(\alpha, \beta) = (0, 1)$ we only consider action or state occupancy, respectively. The case $(\alpha, \beta) = (0, 1)$ is understood as the limit case where α becomes infinitely small. We note that the case $(\alpha, \beta) = (1, 0)$ has often been used along with an external reward with the aim of regularizing the external reward objective [46, 47, 49, 50, 52]. We also note that the case $(\alpha, \beta) = (1, -1)$, with negative β, constitutes an approximation to empowerment [20, 73]: the agent tries to maximize action entropy while minimizing state entropy conditioned to the previous action-state, which favors paths where there is more control on the resulting states. However, we do not consider this case in this paper.

Under the more general intrinsic reward, the expected return obeys

$$V_\pi(s) = \sum_{a,s'} \pi(a|s)p(s'|s,a)\left(-\ln \pi^\alpha(a|s)p^\beta(s'|s,a) + \gamma V_\pi(s')\right). \tag{B.3}$$

Our goal is to maximize the expected return over the policy probabilities $\pi = \{\pi(a|s) : a \in A(s), s \in S\}$ to obtain the optimal policy. Note that for $\alpha > 0$ and $\beta \geq 0$ the expected return is non-negative, $V_\pi(s) \geq 0$.

Theorem 2. *The critical values $V^c(s)$ of the expected returns $V_\pi(s)$ in equation (B.3) with respect to the policy probabilities $\pi = \{\pi(a|s) : a \in A(s), s \in S\}$ obey*

$$V^c(s) = \alpha \ln Z(s) = \alpha \ln\left[\sum_{a\in\mathcal{A}(s)} \exp\left(\alpha^{-1}\beta\mathcal{H}(S'|s,a) + \alpha^{-1}\gamma\sum_{s'} p(s'|s,a)V^c(s')\right)\right] \tag{B.4}$$

where $\mathcal{H}(S'|s,a) = -\sum_{s'} p(s'|s,a)\ln p(s'|s,a)$ is the entropy of the successors of s after performing action a, and $Z(s)$ is the partition function.

The critical points (critical policies) are

$$\pi^c(a|s) = \frac{1}{Z(s)}\exp\left(\alpha^{-1}\beta\mathcal{H}(S'|s,a) + \alpha^{-1}\gamma\sum_{s'} p(s'|s,a)V^c(s')\right), \tag{B.5}$$

one per critical value, where the partition function $Z(s)$ is the normalization constant.

Defining $z_i = \exp\big(\alpha^{-1}\gamma V^c(s_i)\big)$, $p_{ijk} = p(s_j|s_i,a_k)$ and $\mathcal{H}_{ik} = \alpha^{-1}\beta\mathcal{H}(S'|s_i,a_k)$, Eq. (B.4) can be compactly rewritten as

$$z_i^{\gamma^{-1}} = \sum_k w_{ik} e^{\mathcal{H}_{ik}} \prod_j z_j^{p_{ijk}} \tag{B.6}$$

where the matrix with coefficients $w_{ik} \in \{0,1\}$ indicates whether action a_k is available at state s_i ($w_{ik} = 1$) or not ($w_{ik} = 0$), and j extends over all states, with the understanding that if a state s_j is not a possible successor from state s_i and action a_k then $p_{ijk} = 0$.

Note that the we simultaneously optimize $|S|$ expected returns, one per state s, each with respect to the set of probabilities $\pi = \{\pi(a|s) : a \in A(s), s \in S\}$.

Proof. We first note that the expected return in Eq. (2) is continuous and has continuous derivatives with respect to the policy except at the boundaries (i.e., $\pi(a|s) = 0$ for some action-state (a,s)). Choosing a state s, we first take partial derivatives with respect to $\pi(a|s)$ for each $a \in \mathcal{A}(s)$ in both sides of Eq. (B.3), and then evaluate them at a critical point π^c to obtain the condition

$$\begin{aligned}\lambda(s,s) &= \sum_{s'} p(s'|s,a)\left(-\ln(\pi^c(a|s))^\alpha p^\beta(s'|s,a) + \gamma V^c(s')\right) - \alpha + \gamma\sum_{b,s'}\pi^c(b|s)p(s'|s,b)\lambda(s',s) \\ &= -\alpha\ln\pi^c(a|s) - \beta\sum_{s'} p(s'|s,a)\ln p(s'|s,a) - \alpha \\ &\quad +\gamma\sum_{s'} p(s'|s,a)V^c(s') + \gamma\sum_{b,s'}\pi^c(b|s)p(s'|s,b)\lambda(s',s),\end{aligned} \tag{B.7}$$

where we have defined the partial derivative at the critical point $\frac{\partial V_\pi(s')}{\partial\pi(a|s)}|_{\pi^c} \equiv \lambda(s',s)$ and used the fact that this partial derivative should be action-independent. To understand this, note that the critical policy should lie in the simplex $\sum_a \pi(a|s) = 1$, $\pi(a|s) \geq 0$, and therefore the gradient of $V_\pi(s')$ with respect to the $\pi(a|s)$ at the critical policy should be along the normal to the constraint surface, i.e., the diagonal direction (hence, action-independent), or be zero. Indeed, the action-independence of the $\lambda(s',s)$ also

results from interpreting them as Lagrange multipliers: $\lambda(s', s)$ is the Lagrange multiplier corresponding to the state-value function at s', $V_\pi(s')$, associated to the constraint $\sum_a \pi(a|s) = 1$, $\pi(a|s) \geq 0$, defining the simplex where the probabilities $\{\pi(a|s) : a \in A(s)\}$ lie.

Noticing that the last term of Eq. (B.7) does not depend on a, we can solve for the critical policy $\pi^c(a|s)$ to obtain equation (B.5). Eq. (B.5) implicitly relates the critical policy with the critical value of the expected returns from each state s. Inserting the critical policy (B.5) into Eq. (B.3), we get (B.4), which is an implicit non-linear system of equations exclusively depending on the critical values.

It is easy to verify that the partial derivatives of $V_\pi(s)$ in Eq. (B.3) with respect to $\pi(a'|s')$ for $s \neq s'$ are

$$\lambda(s, s') = \gamma \sum_{s''} p(s''|s)\lambda(s'', s'),$$

and thus they provide no additional constraint on the critical policy. [1]

□

We finally show that the optimal expected returns, as defined from the Bellman optimality equation

$$V^*(s) = \max_{\pi(\cdot|s)} \sum_{a,s'} \pi(a|s)p(s'|s,a) \left(-\ln \pi^\alpha(a|s)p^\beta(s'|s,a) + \gamma V^*(s') \right), \tag{B.8}$$

obey the same Eq. (B.4) as the critical values of Eq. (B.3) do. To see this, note that after taking partial derivatives with respect to $\pi(a|s)$ for each $a \in \mathcal{A}(s)$ on the right-hand side of Eq. (B.8) we get

$$0 = -\alpha \ln \pi(a|s) - \beta \sum_{s'} p(s'|s,a) \ln p(s'|s,a) + \gamma \sum_{s'} p(s'|s,a)V^*(s') - \alpha + \lambda(s), \tag{B.9}$$

where $\lambda(s)$ is the Lagrange multiplier associated to the constraint $\sum_a \pi(a|s) = 1$. This equation, except for the irrelevant action-independent Lagrange multipliers, is identical to Eq. (B.7). Eq. (B.4) follows from inserting the resulting optimal policy into the Bellman optimality equation.

C Unicity of the optimal value and policy, and convergence of the algorithm

We now prove that the critical value $V^c(s)$ is unique, in other words, equation (B.4) admits a single solution (Theorem 3). We later prove that the solution is the optimal expected return (Theorem 4).

Theorem 3. *With the definitions in Theorem 2, the system of equations*

$$z_i^{\gamma^{-1}} = \sum_k w_{ik} e^{\mathcal{H}_{ik}} \prod_j z_j^{p_{ijk}} \tag{C.1}$$

with $0 < \gamma < 1$*,* $\alpha > 0$ *and* $\beta \geq 0$ *has a unique solution in the positive first orthant* $z_i > 0$*, provided that for all* i *there exists at least one* k *such that* $w_{ik} = 1$*. The solution satisfies* $z_i \geq 1$*.*

Moreover, given any initial condition $z_i^{(0)} > 0$ *for all* i*, the infinite series* $z_i^{(n)}$ *defined through the iterative map*

[1] This set of equations along with Eq. (B.7) generates a linear system of $\mathcal{S}^2$ equations for the $\mathcal{S}^2$ unknowns $\lambda(s, s')$. In the next section we show that the critical values $V^c(s)$ and critical policy $\pi^c(a|s)$ exists and are unique, and thus the system of equations for $\lambda(s, s')$ is of the type $\Lambda = \gamma P^\intercal \Lambda + F$, with unique matrices $\Lambda_{ss'} = \lambda(s, s')$, $P_{s's} = p(s'|s) \equiv \sum_a \pi^c(a|s)p(s'|s,a)$ and $F_{s's}$ is a diagonal matrix with $F_{ss} = V^c(s) - \alpha$. Because P is a stochastic matrix, it does not have eigenvalues larger than one. Therefore the matrix $\mathbb{I} - \gamma P^\intercal$ with $\gamma < 1$ does not have zero eigenvalues, and thus it is invertible. The solution to the system is then unique and given thenby $\Lambda = (\mathbb{I} - \gamma P^\intercal)^{-1} F$.

$$z_i^{(n+1)} = \left(\sum_k w_{ik} e^{\mathcal{H}_{ik}} \prod_j \left(z_j^{(n)} \right)^{p_{ijk}} \right)^{\gamma} \tag{C.2}$$

for $n \geq 0$ converges to a finite limit $z_i^{\infty} \geq 1$, and this limit is the unique solution of equation (C.1)

Note that the condition that for all i there exists at least one k such that $w_{ik} = 1$ imposes virtually no restriction, as it only asks for the presence of at least one available action in each state. For instance, in absorbing states, the action leads to the same state.

Importantly, proving that the map (C.2) has a single limit regardless of the initial condition in the positive first orthant $z_i^{(0)} > 0$ suffices to prove that equation (C.1) has a unique solution in that region, as then no other fix point of the map can exist. Additionally, since the solution is unique and satisfies $z_i^{\infty} \geq 1$, the critical state-value function that solves equation (B.4) is unique, and $V^c(s_i) = \alpha\gamma^{-1} \ln z_i^{\infty} \geq 0$, consistent with its properties.

The map (C.2) provides a useful value-iteration algorithm used in examples shown in the Results section, and empirically is found to rapidly converge to the solution.

Proof. We call the series $z_i^{(n)}$ with initial condition $z_i^{(0)} = 1$ for all i the *main* series. We first show that the main series is monotonic non-decreasing.

For $n = 1$, we get

$$z_i^{(1)} = \left(\sum_k w_{ik} e^{\mathcal{H}_{ik}} \prod_j (1)^{p_{ijk}} \right)^{\gamma} \geq 1 = z_i^{(0)} \tag{C.3}$$

for all i, using that there exists k for which, $w_{ik} = 1$, w_{ik} is non-negative for all i and k, $\mathcal{H}_{ik} \geq 0$ and the power function x^{γ} is increasing with its argument.

Assume that for some $n > 0$, $z_i^{(n)} \geq z_i^{(n-1)}$ for all i. Then

$$z_i^{(n+1)} = \left(\sum_k w_{ik} e^{\mathcal{H}_{ik}} \prod_j \left(z_j^{(n)} \right)^{p_{ijk}} \right)^{\gamma} \geq \left(\sum_k w_{ik} e^{\mathcal{H}_{ik}} \prod_j \left(z_j^{(n-1)} \right)^{p_{ijk}} \right)^{\gamma} = z_i^{(n)} \tag{C.4}$$

using the same properties as before, which proves the assertion for all n by induction.

Now let us show that the main series is bounded. Define $\mathcal{H}_{\text{max}} = \max_{ik} \mathcal{H}_{ik}$, and obviously $\mathcal{H}_{\text{max}} \geq 0$.

For $n = 1$ we have

$$z_i^{(1)} = \left(\sum_k w_{ik} e^{\mathcal{H}_{ik}} \right)^{\gamma} \leq \left(|A|_{\text{max}} e^{\mathcal{H}_{\text{max}}} \right)^{\gamma} \equiv c^{\gamma} \tag{C.5}$$

(remember that $|A|_{\text{max}}$ is the maximum number of available actions from any state).

For $n = 2$,

$$\begin{aligned} z_i^{(2)} &= \left(\sum_k w_{ik} e^{\mathcal{H}_{ik}} \prod_j \left(z_j^{(1)} \right)^{p_{ijk}} \right)^{\gamma} \leq \left(\sum_k w_{ik} e^{\mathcal{H}_{ik}} \prod_j c^{\gamma p_{ijk}} \right)^{\gamma} \\ &= \left(\sum_k w_{ik} e^{\mathcal{H}_{ik}} c^{\gamma} \right)^{\gamma} = c^{\gamma^2} \left(\sum_k w_{ik} e^{\mathcal{H}_{ik}} \right)^{\gamma} \leq c^{\gamma + \gamma^2} \end{aligned}$$

using the standard properties, $\sum_j p_{ijk} = 1$ and Eq. (C.5).

Assume that for some $n > 1$ we have $z_i^{(n)} \leq c^{\gamma + \gamma^2 + \ldots + \gamma^n}$. We have just showed that this is true for $n = 2$. Then

$$
\begin{aligned}
z_i^{(n+1)} &= \left(\sum_k w_{ik} e^{\mathcal{H}_{ik}} \prod_j \left(z_j^{(n)}\right)^{p_{ijk}}\right)^\gamma \le \left(\sum_k w_{ik} e^{\mathcal{H}_{ik}} c^{\gamma+\ldots+\gamma^n}\right)^\gamma \\
&= c^{\gamma^2+\ldots+\gamma^{n+1}} \left(\sum_k w_{ik} e^{\mathcal{H}_{ik}}\right)^\gamma \le c^{\gamma+\ldots+\gamma^{n+1}}
\end{aligned}
$$

and therefore it is true for all $n \ge 0$ by induction.

Therefore the series $z_i^{(n)}$ is bounded by $c^{1/(1-\gamma)}$. Together with the monotonicity of the series, we have now proved that the limit z_i^∞ of the series exists. Moreover, $z_i^\infty \ge z_i^0 = 1$.

The above results can be intuitively understood: the 'all ones' initial condition of the main series corresponds to an initial guess of the state-value function equal to zero everywhere. The iterative map corresponds to state-value iteration to a more optimistic value: as intrinsic reward based on entropy is always non-negative, the z-values monotonically increase after every iteration. Finally, the z-values reach a limit because the state-value function is bounded.

We now show the central result that the series obtained by using the iterative map starting from any initial condition in the positive first orthant can be bounded below and above by two series that converge to the main series. Therefore, by building 'sandwich' series we will confirm that any other series has the same limit as the main series.

Let the $y_i^{(0)} = u_i > 0$ be the initial condition of the series $y_i^{(n)}$ obeying the iterative map (C.2), and define $u_{\text{min}} = \min_i u_i$ and $u_{\text{max}} = \max_i u_i$. Obviously, $u_{\text{min}} > 0$ and $u_{\text{max}} > 0$. Applying the iterative map once, we get

$$
\begin{aligned}
y_i^{(1)} &= \left(\sum_k w_{ik} e^{\mathcal{H}_{ik}} \prod_j \left(y_j^{(0)}\right)^{p_{ijk}}\right)^\gamma \le \left(\sum_k w_{ik} e^{\mathcal{H}_{ik}} \prod_j (u_{\text{max}})^{p_{ijk}}\right)^\gamma \\
&= \left(\sum_k w_{ik} e^{\mathcal{H}_{ik}} u_{\text{max}}\right)^\gamma = u_{\text{max}}^\gamma \left(\sum_k w_{ik} e^{\mathcal{H}_{ik}}\right)^\gamma = u_{\text{max}}^\gamma z_i^{(1)}
\end{aligned}
$$

where in the last step we have used the values of the main series in the first iteration. We can similarly lower-bound $y_i^{(1)}$ to finally show that it is both lower- and upper-bounded by $z_i^{(1)}$ with different multiplicative constants,

$$
u_{\text{min}}^\gamma z_i^{(1)} \le y_i^{(1)} \le u_{\text{max}}^\gamma z_i^{(1)} \tag{C.6}
$$

Now, assume that

$$
u_{\text{min}}^{\gamma^n} z_i^{(n)} \le y_i^{(n)} \le u_{\text{max}}^{\gamma^n} z_i^{(n)} \tag{C.7}
$$

is true for some $n > 0$. Then, for $n+1$ we get

$$
\begin{aligned}
y_i^{(n+1)} &= \left(\sum_k w_{ik} e^{\mathcal{H}_{ik}} \prod_j \left(y_j^{(n)}\right)^{p_{ijk}}\right)^\gamma \le \left(\sum_k w_{ik} e^{\mathcal{H}_{ik}} \prod_j \left(u_{\text{max}}^{\gamma^n} z_i^{(n)}\right)^{p_{ijk}}\right)^\gamma \\
&= u_{\text{max}}^{\gamma^{n+1}} \left(\sum_k w_{ik} e^{\mathcal{H}_{ik}} \prod_j \left(z_i^{(n)}\right)^{p_{ijk}}\right)^\gamma = u_{\text{max}}^{\gamma^{n+1}} z_i^{(n+1)}
\end{aligned}
$$

by simply extracting the common factor in the fourth expression, remembering that $\sum_j p_{ijk} = 1$, and using the definition of the main series in the last one. By repeating the same with the lower bound, we finally find that (C.7) holds also for $n+1$, and then, by induction, for every $n > 0$.

The proof concludes by noticing that the limit of both $u_{\text{max}}^{\gamma^n}$ and $u_{\text{min}}^{\gamma^n}$ is 1, and therefore using (C.7) the limit y_i^∞ of the series $y_i^{(n)}$ equals the limit of the main series, $y_i^\infty = z_i^\infty$.

Note that the iterative map (C.2) is not necessarily contractive in the Euclidean metric, as it is possible that, depending on the values of u_{min} and u_{max} and the changes in the main series, the bounds in Eq. (C.7) initially diverge to finally converge in the limit.

□

Theorem 4. *The (unique) critical value $V^c(s)$ is the optimal expected return, that is, the one that attains the maximum expected return at every state for any policy, and we write $V^c(s) = V^*(s)$*

Proof. To show that $V^c(s)$ is the optimal expected return, we note that the maximum of the functions $V_\pi(s)$ with respect to policy π should be at the critical policy or at the boundaries of the simplices defined by $\sum_a \pi(a|s) = 1$ with $0 \leq \pi(a|s) \leq 1$ for every a and s, as the expected return $V_\pi(s)$ is continuous and has continuous derivatives with respect to the policy except at the boundaries. At the policy boundary, there exists a non-empty subset of states s_i and a non-empty set of actions a_k for which $\pi(a_k|s_i) = 0$. Computing the critical value of the expected return along that policy boundary is identical to moving from the original to a new problem where we replace the graph connectivity matrix w_{ik} in Eq. (C.1) by a new one v_{ik} such that $v_{ik} \leq w_{ik}$ (remember that at the boundary there should be an action a_k that were initially available from state s_i, $w_{ik} = 1$, that at the policy boundary is forbidden, $v_{ik} = 0$). We now define the convergent series $z_i^{(n)}$ and $y_i^{(n)}$ for the original and new problems respectively by using the iterative map (C.2) with initial conditions equal to all ones. We prove now that $z_i^{(n)} \geq y_i^{(n)}$ for all i for $n = 1, 2, ...$, and thus their limits obey $z_i^\infty \geq y_i^\infty$.

For $n = 1$, we get

$$z_i^{(1)} = \left(\sum_k w_{ik} e^{\mathcal{H}_{ik}} \prod_j (1)^{p_{ijk}} \right)^\gamma \geq \left(\sum_k v_{ik} e^{\mathcal{H}_{ik}} \prod_j (1)^{p_{ijk}} \right)^\gamma = y_i^{(1)} \tag{C.8}$$

for all i, using that $w_{ik} \geq v_{ik}$ and that the power function x^γ is increasing with its argument.

Assuming that $z_i^{(n)} \geq y_i^{(n)}$ for all i for some $n > 0$, then

$$z_i^{(n+1)} = \left(\sum_k w_{ik} e^{\mathcal{H}_{ik}} \prod_j \left(z_j^{(n)} \right)^{p_{ijk}} \right)^\gamma \geq \left(\sum_k v_{ik} e^{\mathcal{H}_{ik}} \prod_j \left(y_j^{(n)} \right)^{p_{ijk}} \right)^\gamma = y_i^{(n+1)} \tag{C.9}$$

using the same properties as before, which proves the assertion for all n by induction.

Remembering that the expected return $V(s_i)$ is increasing with z_i, we conclude that the expected return obtained from policies restricted on the boundaries of the simplices is no better than the original critical value of the expected return.

□

D Particular examples

Here we summarize the main results and specialize them to specific cases. We assume $0 < \gamma < 1$, $\alpha > 0$ and $\beta \geq 0$ and use the notation $z_i = \exp\big(\alpha^{-1}\gamma V^*(s_i)\big)$, where $V^*(s)$ is the optimal expected return, $p_{ijk} = p(s_j|s_i, a_k)$ and $\mathcal{H}_{ik} = \alpha^{-1}\beta\mathcal{H}(S'|s_i, a_k)$, where $\mathcal{H}(S'|s, a) = -\sum_{s'} p(s'|s, a) \ln p(s'|s, a)$.

D.1 Action-state entropy maximizers

Agents that seek to maximize the discounted action-state path entropy follow the optimal policy

$$\pi^*(a_k|s_i) = \frac{1}{Z_i} \left(w_{ik} e^{\mathcal{H}_{ik}} \prod_j z_j^{p_{ijk}} \right) \tag{D.1}$$

with

$$Z_i = \sum_k w_{ik} e^{\mathcal{H}_{ik}} \prod_j z_j^{p_{ijk'}} \tag{D.2}$$

The matrix with coefficients $w_{ik} \in \{0, 1\}$ indicate whether action a_k is available at state s_i ($w_{ik} = 1$) or not ($w_{ik} = 0$).

The expected return (state-value function) in terms of the z variables obeys

$$z_i^{\gamma^{-1}} = \sum_k w_{ik} e^{\mathcal{H}_{ik}} \prod_j z_j^{p_{ijk}} \tag{D.3}$$

D.2 Action-only entropy maximizers

Agents that ought to maximize the time-discounted action path entropy correspond to the above case with $\beta = 0$, and therefore the optimal policy reads as

$$\pi^*(a_k|s_i) = \frac{1}{Z_i} \left(w_{ik} \prod_j z_j^{p_{ijk}} \right) \tag{D.4}$$

with

$$Z_i = \sum_k w_{ik} \prod_j z_j^{p_{ijk}} \tag{D.5}$$

The state-value function in terms of the z variables obeys

$$z_i^{\gamma^{-1}} = \sum_k w_{ik} \prod_j z_j^{p_{ijk}} \tag{D.6}$$

D.3 Entropy maximizers in deterministic environments

In a deterministic environment $p_{i,j(i,k),k} = 1$ for successor state $j = j(i, k)$, and zero otherwise. In this case, at every state i we can identify an action k with its successor state j. Therefore, the optimal policy is

$$\pi^*(a_k|s_i) = \frac{w_{ij} z_j}{Z_i} \tag{D.7}$$

with

$$Z_i = \sum_j w_{ij} z_j \tag{D.8}$$

The state-value function in terms of the z variables reads

$$z_i^{\gamma^{-1}} = \sum_j w_{ij} z_j \tag{D.9}$$

E Experiments

In this subsection, we present the details for the numerical simulations performed for the different experiments in the manuscript. First, we discuss the construction of the MOP and R agents, and afterwards we present the details of each particular experiment.

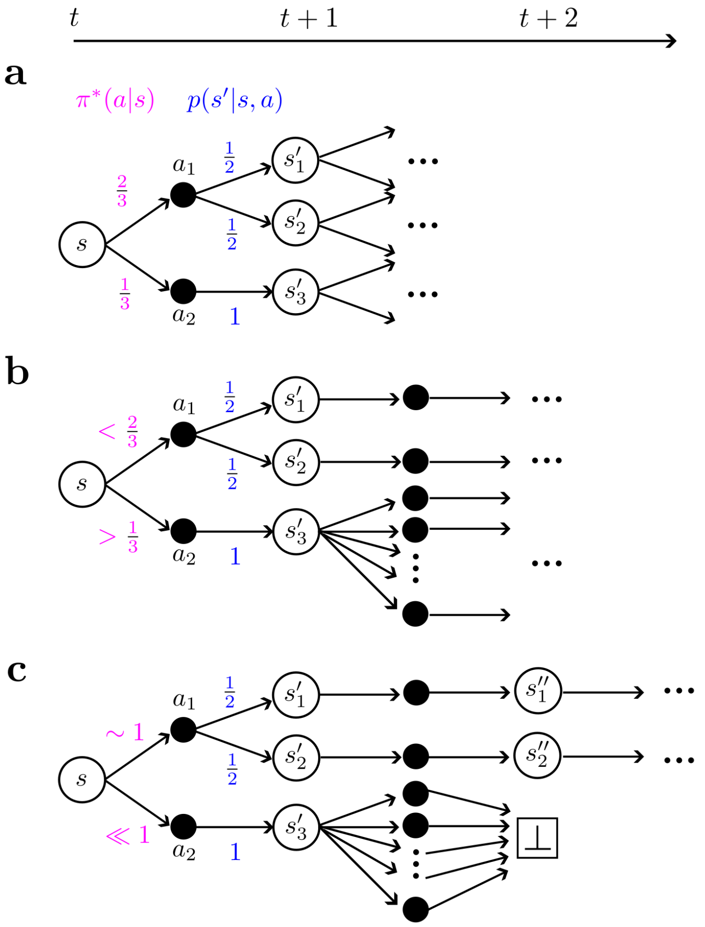

Figure D.1: MOP agents determine stochastic policies that maximize occupancy of future action-state paths. In all panels, the three successive dots indicate that the future looks the same for all the states or actions involved from that point onwards. (a) At time t, the agent is faced with determining the optimal policy at state s. Given that taking action a_1 can stochastically lead to two distinct states s'_1 and s'_2, the optimal policy gives action a_1 twice the probability weight than to action a_2 (which only induces a deterministic transition to state s'_3). From time $t+1$, the future looks the same from all three states s'_i. (b) If the future does not look the same, and actually there are many more actions available at state s'_3 compared to s'_1 and s'_2, then more weight should be given to action a_2 than if the future was the same. (c) If, however, all the actions available at state s'_3 lead you to an absorbing state, almost zero weight should be given to action a_2.

E.1 MOP agent

In all the experiments presented, we introduce the MOP agent. The objective function that this agent maximizes in general is Eq. (2). As described in section D, the α and β parameters control the weights of action and next-state entropies to the objective function, respectively. Unless indicated otherwise, we always use $\alpha = 1, \beta = 0$ for the experiments. It is important to note, as we have done before, that if the environment is deterministic, then the next-state entropy $\mathcal{H}(S'|s,a) = -\sum_{s'} p(s'|s,a) \ln p(s'|s,a) = 0$, and therefore β does not change the optimal policy, Eq. (6).

We have implemented the iterative map, Eq. (7), to solve for the optimal value, using $z_i^{(0)} = 1$ for all i as initial condition. Theorem (3) ensures that this iterative map finds a unique optimal value regardless of the initial condition in the first orthant. To determine a degree of convergence, we compute the supremum norm between iterations,

$$\delta = \max_i |V_i^{(n+1)} - V_i^{(n)}|,$$

where $V_i = \frac{\alpha}{\gamma} \log(z_i)$, and the iterative map stops when $\delta < 10^{-3}$.

E.2 R agent

We also introduce a reward-maximizing agent in the usual RL sense. In this case, the reward is $r = 1$ for living and $r = 0$ when dying. In other words, this agent maximizes life expectancy. Additionally, to emphasize the typical reward-seeking behavior and avoid degenerate cases induced by the tasks, we introduced a small reward for the Four-room grid world (see below). In all other aspects, the modelling of the R agent is identical to the MOP agent. To allow for reward-maximizing agents to display some stochasticity, we used an ϵ-greedy policy, the best in the family of ϵ-soft policies [14]. At any given state, a random admissible action is chosen with probability ϵ, and the action that maximizes the value is chosen with probability $1 - \epsilon$. Given that the world models $p(s'|s, a)$ are known and the environments are static, this ϵ-greedy policy does not serve the purpose of exploration (in the sense of learning), but only to inject behavioral variability. Therefore, we construct an agent with state-independent variability, whose value function satisfies the optimality Bellman equation for this ϵ-greedy policy,

$$V_\epsilon(s) = (1-\epsilon)\max_a \sum_{s'} p(s'|s,a)\left(r + \gamma V_\epsilon(s')\right) + \frac{\epsilon}{|\mathcal{A}(s)|}\sum_{a,s'} p(s'|s,a)\left(r + \gamma V_\epsilon(s')\right), \tag{E.1}$$

where $|\mathcal{A}(s)|$ is the number of admissible actions at state s. To solve for the optimal value in this Bellman equation, we perform value iteration [14]. The ϵ-greedy policy for the R agent is therefore given by

$$\pi(a|s) = \begin{cases} 1 - \epsilon + \frac{\epsilon}{|\mathcal{A}(s)|}, & \text{if } a = \arg\max_{a'} \sum_{s'} p(s'|s,a')\left(r + \gamma V_\epsilon(s')\right) \\ \frac{\epsilon}{|\mathcal{A}(s)|}, & \text{otherwise} \end{cases}$$

where ties in $\arg\max$ are broken randomly. Note that if $\epsilon = 0$, we obtain the usual greedy optimal policy that maximizes reward.

E.3 Four-room grid world

Environment The arena is composed of four rooms, each having size 5×5 locations where the agent can be in. From each room, the agent can go to two adjacent rooms through small openings, each located in the middle of the wall that separates the rooms. At each of these rooms, there is a food source located in the corner furthest from the openings. See Fig. 2 for a graphic description. Unless indicated otherwise, the discount factor is set to $\gamma = 0.99$.

States The states are the Cartesian product between (x, y) location and internal state u, which is simply a scalar value between a minimum of 0 and a maximum capacity of 100. All states such that $(x, y, u = 0)$ are absorbing states, independently of the location (x, y). The particular internal state $u = 100$ is the maximum capacity for energy, such that even when at a food source, this internal state does not change. Therefore, the number of states in this experiment is $|\mathcal{S}| = 104\,\text{external states} \times 101\,\text{internal states} = 10504$.

Actions The agent has a maximum of 9 actions: `up, down, left, right, up left, up right, down left, down right`, and `nothing`. Whenever the agent is close to a wall, the number of available actions decreases such that the agent cannot choose to go into walls. Finally, whenever the agent is in an absorbing state, only `nothing` is available.

Transitions At any transition, there is a cost of 1 unit of energy for being alive. On the other hand, whenever the agent is located at a food source, there is an increase in energy that we vary parametrically that we call food gain g. For example, if the agent is in location $(2, 1)$ at time t and moves towards $(1, 1)$ (where food is located), the change in energy would be $\Delta u_t = -1$, given that the change in internal energy depends only on the current state and action. If the agent decides to stay in $(1, 1)$ at time $t + 1$, then $\Delta u_{t+1} = -1 + g$.

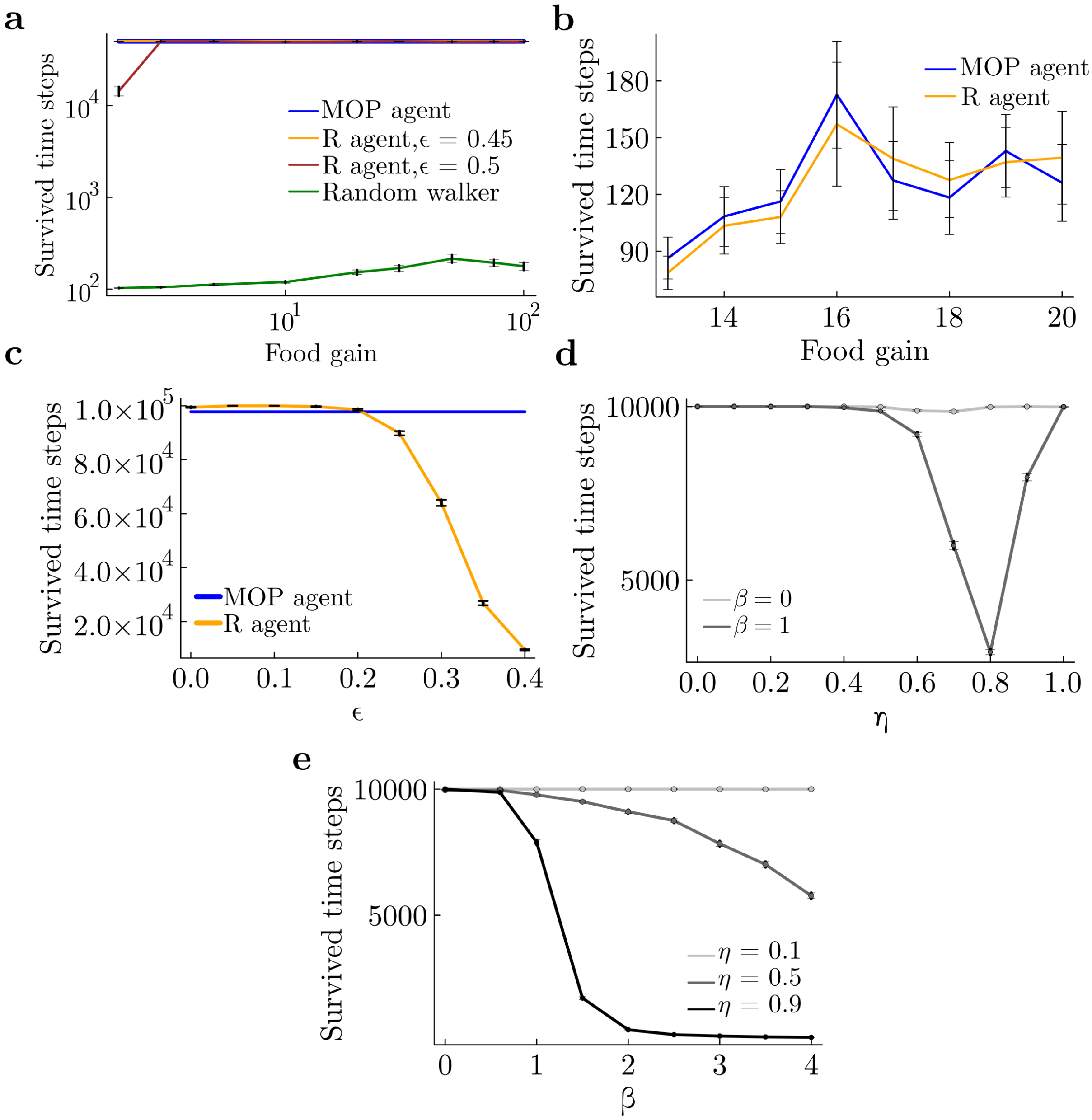

Figure E.2: Survivability for the experiments considered in the manuscript. (a) Survivability of the various agents tested in the four-room grid world. At each 5E4 timestep episode, we recorded the survived time and averaged across episodes. (b) Survivability of the mouse for both MOP and R agents. (c) Survivability for the cartpole (Sec. E.5) in the deterministic arena for the MOP agent and the ϵ-greedy R agents, $\gamma = 0.98$. (d) Survivability for cartpole (Sec. E.5) in the stochastic arena for the $\beta = 0$ and the $\beta = 1$ MOP agents. $\gamma = 0.99$. (e) Survivability of the cartpole (Sec. E.5) MOP agents as a function of β, for various values of η. $\gamma = 0.99$

R agent As stated above, in this experiment we introduced an extra reward for the R agent when it reaches the food source. The magnitude is small compared to the survival reward ($1E - 5$ smaller) and it mainly serves to break the degeneracy of the value function. The variability of the R agent is thus coming purely from the ϵ-greedy action selection.

Survivability To allow for the maximum uniform variability for the R agent, we tested various values for ϵ and observed the survivability of the agents as a function of ϵ, across all the food gains tested (see Results section). The value of ϵ for which the R agent still survives as much as the MOP agent is $\epsilon = 0.45$ (see Figure E.2a).

Noisy room In this variation for the experiment, there is a room (the bottom right room) where transitions are uniformly random for all actions, across all possible neighboring locations. That is, for any location s_{nr} in the noisy room, and any a available at that location, given that it has $n(s_{nr})$ total neighbours (including the same location),

$$p(s'|s_{nr}, a) = \begin{cases} \frac{1}{n(s_{nr})} & \text{for } s' \in \text{neighbours} \\ 0 & \text{otherwise} \end{cases}$$

E.4 Predator-prey scenario

Here we provide all details of the simulated experiments. Results are shown in Fig. 3.

Environment The environment is similar to that one used for the 4-room grid world described in E.3. Apart from the agent (prey), there is also another moving subject (predator) with a simple predefined policy. The grid world consists of a "home" area, a rectangle 2x3 where the agent may enter, but the predator cannot. This home area has a small opening that leads to a bigger 4x7 rectangle arena available for both the agent and the predator. The only food source is located at the bottom-right corner of the common part of the arena, so that the agent needs to leave its home to boost its energy. Additionally, there is an obstacle which separates the arena in two parts with two openings, above and under the obstacle. This obstacle allows the agent to "hide" from the predator behind it.

States The location of the predator is part of the agent's state, such that a particular state consists of the position of the agent, the position of the predator and the amount of energy of the agent. For this case, we set the maximum amount of energy F equal to the food gain. Positions are 2-dimensional, and therefore the states are 5-dimensional. In the used arena there are 33 possible locations for the agent and 26 ones for the predator, so that the total number of states ranges from 11154 for $F = 13$ to 17160 for $F = 20$.

Actions The agent has the same actions as in the four-room grid world. The maximum number of available actions is therefore 9. Moving towards obstacles or walls is not allowed.

Transitions The agent loses one unit of energy every time step and increases the amount of energy up to a given maximum capacity level F only at the food source. If the position of both the agent and the predator are the same, then the agent is "eaten" and moves to the absorbing state of death as well as in the case of energy equal to 0. After entering the absorbing state the agent stays there forever.

The predator also moves as the agent (horizontally, vertically, diagonally on one step or to stay still). Steps of the agent and the predator happen synchronously. The predator is "attracted" to the agent: the probability of moving to some direction is an increasing function on the cosines $\cos \alpha_k$ of the angle α_k between this direction of motion k and the direction of the radius vector from the predator to the agent. In particular, this probability is

$$p_k^c = C^{-1} \exp(\kappa \cos \alpha_k) \quad \text{(E.2)}$$

where κ is the inverse temperature of the predator and $C = \sum_k \exp(\kappa \cos \alpha_k)$ is a normalization factor. These probabilities are computed only for motions available at the current location of the predator, so that e.g. for the location at the wall the motions along the wall are taken into account, but not the motion towards the wall.

Goal The goal of the MOP agent is to maximize discounted action entropy, and thus to find the optimal state-value function using the iterative map in Eq. (7) with $\mathcal{H}_{ik} = 0$ ($\beta = 0$). While using the iterative map, we take advantage of the fact that given an action the physical transition of the agent is deterministic, but the physical transition of the predator is stochastic. Therefore, the sum over successor states j in Eq. (7) is simply a sum over the predator successor states.

Parameters $\gamma = 0.98$, $F = 15$ (if another value between 13 and 20 not mentioned), $\kappa = 2$. Simulation time is 5000 steps.

Counting rotations We define a clockwise (counterclockwise) half-rotation as the event when the agent came from the left part of the arena to the right part over the field above (under) the wall and from the right part to the left one over the field under (above) the wall without crossing the vertical line of the wall in between. One full rotation consists of two half-rotations in the same directions performed one after another. We counted the number of full rotations in both directions in 70 episodes of 500 time steps each for both MOP and R agents for different values of the food gain F. Error bars were computed based on these 70 repetitions. The fraction of clockwise rotations to total rotations (sum of clockwise and anticlockwise rotations) for different values of F is shown at Fig. 3.

Survivability The ϵ-greedy R agents display some variability that depends on ϵ. To select this parameter, we matched average lifetimes (measured in simulations of 5000 steps length) between the MOP and R agents, separately for every F. Lifetimes are plotted in Figure E.2b.

Videos We have generated one video for the MOP agent (Video 2) and another for the R agent (Video 3), both for $F = 15$, $\kappa = 2$, and $\epsilon = 0.06$ for the R agent so as to match their average lifetimes as described above. In the videos, green vertical bar indicates the amount of energy by the agent at current time. When the agent makes at least one full rotation around the wall, it is indicated by the written phrase "clockwise rotation" or "anticlockwise rotation". Black vertical arrow indicates direction ('up' for clockwise and 'down' for anticlockwise directions) of the half-rotation in the part of arena left from the wall.

E.5 Cartpole

Environment A cart is placed in a one-dimensional track with boundaries at $|x| = 1.8$. It has a pole attached to it, that rotates like an inverted pendulum with its pivot point on the cart.

States The dynamical system can be described by a four-dimensional external state (x, v, θ, ω), where x is the position of the cart, v is its linear velocity, θ is the angle of the pole with respect to the vertical which grows counterclockwise, and ω is its angular velocity. In this case, we model the internal state u simply with the binary variable `alive, dead`, where the agent enters the absorbing state `dead` if its position exceeds the boundaries, or if its angle exceeds 36 degrees. This amplitude of angles is larger than that typically assumed (12 degrees in [85]), and therefore our system is allowed to be more non-linear and unstable. The state space is $[-1.8, 1.8] \times (-\infty, \infty) \times [-36, 36] \times (-\infty, \infty) \times \{0, 1\}$. To solve for the state value function in Eq. (7), we discretize the state space by setting a maximum value for the velocities. Given all the parameters (allowed x and θ, magnitude of the forces, masses of cart and pole, length of pole and gravity, below), we empirically set the maximum values for $|v| = 6$ and $|\omega| = 3$, which the cart

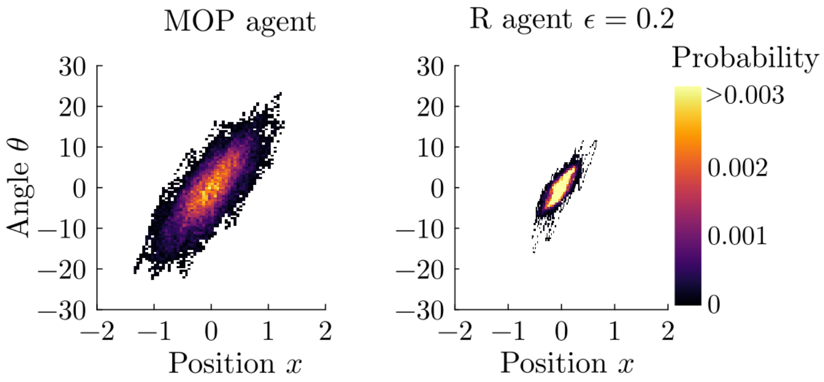

Figure E.3: Histogram of angles and locations visited for the cartpole, as in Fig. 4 of the main manuscript, for the MOP agent (left) and ϵ-greedy R agent (right), with ϵ chosen such that MOP and R agents' lifetimes are similar (see Fig. E.2c).

actually never exceeds. Therefore, we computed the state value function in a $31 \times 31 \times 31 \times 31 \times 2$ grid (number of states = 1.8×10^6).

Actions Any time the agent is `alive`, it has 5 possible actions: forces of $\{-40, -10, 0, 10, 40\}$, where zero force is understood as `nothing`. If the agent is `dead`, then only `nothing` is allowed.

Transitions This dynamical system is a standard task in reinforcement learning, namely the `cartpole-v0` system of the OpenAI gym [85]. The solution of this dynamical system is given in Ref. [84], where we use a frictionless cartpole. The equations for angular and linear accelerations are thus

$$\ddot{\theta} = \frac{-g\sin(\theta) + \frac{\cos(\theta)}{M+m}\left(-F + m\dot{\theta}^2 l \sin(\theta)\right)}{l\left(\frac{4}{3} - \frac{m\cos^2(\theta)}{M+m}\right)} \tag{E.3}$$

$$\ddot{x} = \frac{1}{\cos(\theta)}\left(\frac{4}{3}l\ddot{\theta} - g\sin(\theta)\right). \tag{E.4}$$

Given a force F, a deterministic transition can be computed from these dynamical rules, and a real-valued state transition is observed by the agents.

R agent The reward signal is 1 each time the agent is alive and 0 otherwise. To allow for some variability in the action selection of the R agent, we implement an ϵ-greedy action selection as described above. For exposition purposes, in the manuscript we set $\epsilon = 0.0$, but we also compared to an R agent with ϵ chosen such that average lifetimes between MOP and R agents are matched (see Fig. E.2c and Fig. E.3).

Parameters Mass of the cart $M = 1$, mass of the pole $m = 0.1$, length of the pole $l = 1$, acceleration due to gravity $g = 9.81$, time discretization $\Delta t = 0.02$. Unless specified differently, the discount factor was set to $\gamma = 0.98$.

Value interpolation The observed external state is a continuous four-dimensional variable, so we need to approximate the value function. In order to do so, we simply discretized the state space as described above, and use value iteration as described in Eq. (5) in these grid points by performing a linear value interpolation for the successor states at each iteration. During a particular episode, the observed states might not be the same as the ones in the grid, so in order to compute the optimal policy at these states, we perform the same type of value interpolation as in the value iteration stage.

Stochastic arena We introduced a slight variation to the environment, where the $x > 0$ half of the arena is noisy: agents choose an action (force), but the intended state transition of applying such an action fails with probability η and succeeds with probability $1 - \eta$. This is implemented as follows: given any state-action pair (s, a) for which $x > 0$, there are two possible successor states, one corresponding to the intended action (force) chosen, and the other one corresponding to a zero force action:

$$p(s'|s,a) = \begin{cases} 1, & \text{if } x < 0 \text{ and } s' \leftarrow (s,a) \\ 1-\eta, & \text{if } x > 0 \text{ and } s' \leftarrow (s,a) \\ \eta, & \text{if } x > 0 \text{ and } s' \leftarrow (s,0) \end{cases} \tag{E.5}$$

This stochasticity lets us differentiate between action path occupancy maximizers and action-state path occupancy maximizers by choosing any positive real value of β in Eq. (1), because $\beta > 0$ agents will have a natural tendency to prefer $x > 0$ locations.

E.6 Agent-pet scenario

An agent and a pet move in an arena with degrees of freedom that depend on the actions made by the agent, as explained next in detail.

Environment A 5×5 arena. The middle column of arena can be blocked by a fence, a vertical obstacle that the pet cannot cross. The agent can cross it freely regardless of whether it is open or closed. The agent can open or close the fence by performing the corresponding action when visiting the lever location, at the left bottom corner.

States The system's state consists of the Cartesian product of agent's location, pet's location and binary state of the fence. So, the number of states is 1250. For the sake of simplicity there is no internal states for the energy, and thus there are not absorbing states. The initial states of the agent and pet at the start of each episode are the middle of the second column and the right lower corner of the arena, respectively.

Actions As in Sec. E.4 the agent's actions are movements to one of the 8 neighbour locations as well as staying on the current one. Additionally, if the agent is on the "lever" location, an additional action is available, namely to open or close the fence, depending on its previous state.

Transitions The pet has the same available movements as the agent when the fence is open. The pet performs a random transition to any of the neighbour locations, or stays still, with the same probability. If the agent closes the fence, then the pet can only move on the side where it lies when closed. For simplicity, if the fence is closed by the agent when the pet lies in the middle column, then the pet can only move to the right or left locations such that it will be at one side of the fence in the next time step.

Goal The goal of the MOP agent is to maximize discounted action-state entropy using the iterative map in Eq. (7) with $\alpha = 1$ and $\beta \in [0, 1]$, parameters that measure the weight of action and state entropies, respectively. As in the prey-predator example, we take advantage of the fact that given an action the physical transition of the agent is deterministic, while the physical transition of the pet is stochastic. Thus, the product over successor states j in Eq. (7) is a product over the pet successor states.

Simulation details We ran simulations for several values of β, from 0 to 1 in 0.1 steps, to interpolate between pure action entropy ($\beta = 0$) and action-state entropy ($\beta = 1$). We measured the fraction of time the gate was open using episodes of 2000 steps averaged over 70 simulations for each β, shown in Fig 5. Heat-maps in that figure correspond to the occupation probability by the pet for $\beta = 0$ (left panel) and $\beta = 1$ (right panel) using an episode of 5000 steps.

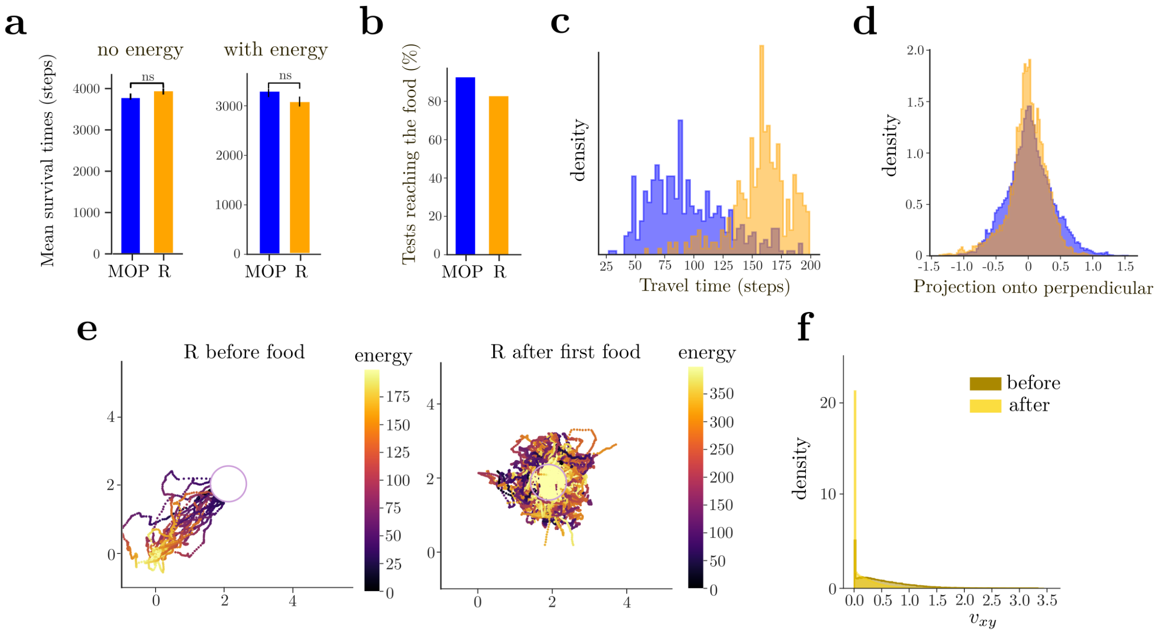

Figure E.4: Comparison between the MOP agent and the R agent in the high-dimensional quadruped (ant) environment from Gymnasium. (a) In both experiments, ϵ for the R agent is chosen as to match the average survival time of the MOP agent. (b) Both MOP and R agents are able to reach the food source in most of the test runs. (c) Probability density function of the travel time, defined as the time the agent spends before encountering the food source for the first time. (d) Probability density function of the projection of all points in a trajectory, for all trajectories, onto the line perpendicular to the shortest path connecting the origin and the food source during travel time (main diagonal). (e) Display of 20 randomly chosen trajectories for the R agent before (left) and after (right) finding the food source for the first time. Colorcode defined by the energy level of the agent. (f) Probability density function of the planar speed before and after finding the food for the first time for the R agent. Both distributions show a peak at very low velocities, indicating prolonged periods of time in which the ant performs very little translational movements.

E.7 Quadruped-Ant

Our goal is to show that MOP also works in high-dimensional, continuous action-state spaces. We employ the Ant-v4 environment from OpenAI's Gymnasium as our testing ground. We benchmark the performance of our entropy-maximizing agent against agents using an ϵ-greedy strategy with rewards $R = 1$ for every step except for absorbing states, where $R = 0$ (R agent). All the relevant hyperparameters used to train these agents are provided in Table 1.

In the first experiment, we study the behavioral variability of our agents and their average lifetime. The agent begins at the (x, y) coordinate $(0, 0)$ and follows its designated policy algorithm. The agent is considered "dead" if either it takes a step that results in the z-coordinate of its torso falling outside the range $[0.3, 1.0]$, or when the episode concludes.

In the second experiment, the agent possesses an energy value, represented as a scalar. The agent dies once it consumes all its energy. Each step taken by the agent consumes one energy point. It commences with an initial energy of 200, and its maximum energy capacity is set at 400. A food source is situated at the $(2, 2)$ coordinate within the arena. Should the agent approach this source within a distance less than 0.5 from the center of its torso, it receives an energy boost of 25. The permissible z-coordinate range for the agent's torso remains consistent with the first experiment. In addition to the state vector provided by the OpenAI Gym environment, we incorporate the agent's energy level, its absolute position, and the food source's position. Interestingly, we found that the MOP agent travels to the food source much faster than the R agent (Supplemental Fig. E.4c, travel time distribution for the MOP agent is shifted towards short times), seemingly appearing less risky, given the stochastic nature of both agents' action selection. Even when the MOP agent travels faster, its trajectories are more variable compared to the

Table 1: Hyperparameters for Ant environment

Parameter	Value
optimizer	Adam
learning rate	3×10^{-4}
discount (γ)	0.999
replay buffer size	10^6
number of hidden layers (all networks)	2
number of hidden units per layer	256
number of samples per minibatch	100
number of training epoch	300
steps per epoch	10000
initial random steps	20000
maximum episode length	5000
nonlinearity	ReLU
target smoothing coefficient (τ)	0.005
number of agents	5
test runs	100

R agent (Supplemental Fig. E.4d, projection of trajectories on a line perpendicular to the straight line that joins the origin and the food source). In this second experiment, we find one out of five R agents not being able to reach the food source in a significant percentage of the test runs. For this reason, we excluded that agent from the analyses.

In the third experiment, the $x > 0$ portion of the arena produces state transition noise. In the unperturbed case (first experiment), given a state s and action a, the agent transitions deterministically to a state $s^p = step(s, a)$ (given by the Gymnasium package). For this experiment, we apply discrete noise to the resulting state s' in the following way: the i-th coordinate of the new state s'_i now independently transitions with probability $1/2$ to either of two states that are close to the unperturbed transition s^p. Specifically,

$$p(s'_i|s,a) = \begin{cases} 1/2 & \text{if} \quad x > 0 \quad \text{and} \quad s'_i = (1+u)s^p_i \\ 1/2 & \text{if} \quad x > 0 \quad \text{and} \quad s'_i = (1-u)s^p_i \\ 1 & \text{if} \quad x \leq 0 \quad \text{and} \quad s'_i = s^p_i \end{cases}, \tag{E.6}$$

where u is the noise magnitude parameter, which makes the transition noisy with probability $1/2$ (note that if $u = 0$, the transition is deterministic, and corresponds to the unperturbed transition). The perturbations thus scale with respect to the unperturbed transition s^p, so that each coordinate gets noise proportional to its magnitude. We apply this noise only to the coordinates given by the 27-dimensional observation vector provided by Gymnasium, so we do not apply noise to the x, y coordinates directly (see details at Ref. [90]). We do not implement an energy constraint in this experiment. The parameter α is set to a constant equal to 1. Note that the intrinsic reward for the next-state transition obtained from being in $x > 0$ is independent of $u > 0$, as $-\beta \log(p(s'|s,a)) = \beta \log 2$, given that the stochasticity of the transition does not depend on the action. Following Eq. (E.6), when $u = 0$, this intrinsic reward vanishes.

We found that $\beta \geq 0$ MOP agents are sensitive to the added noise, and they all managed to survive for almost the whole duration of the episodes after training (Fig. E.5a). First of all, when there is no noise ($u = 0$), the transition is deterministic, and agents do not show any preference to either side of the arena (Fig. E.5b, grey line, c, first row). When noise magnitude is finite but small ($u = 0.01$), $\beta = 0$ MOP agents do not show a significant preference between halves of the arena (Fig. E.5b,c). However, $\beta > 0$ MOP agents show a preference for the half of the arena that produces state transition noise (Fig. E.5b blue line, c second row). If the noise magnitude is larger ($u = 5\%, 7\%$), small β MOP agents (including $\beta = 0$) avoid the noisy half of the arena, given that noise can more easily cause the agent to fall (Fig. E.5b,c). Crucially, with increasing $\beta > 0$ we see an increasing preference for the noisy half of the arena

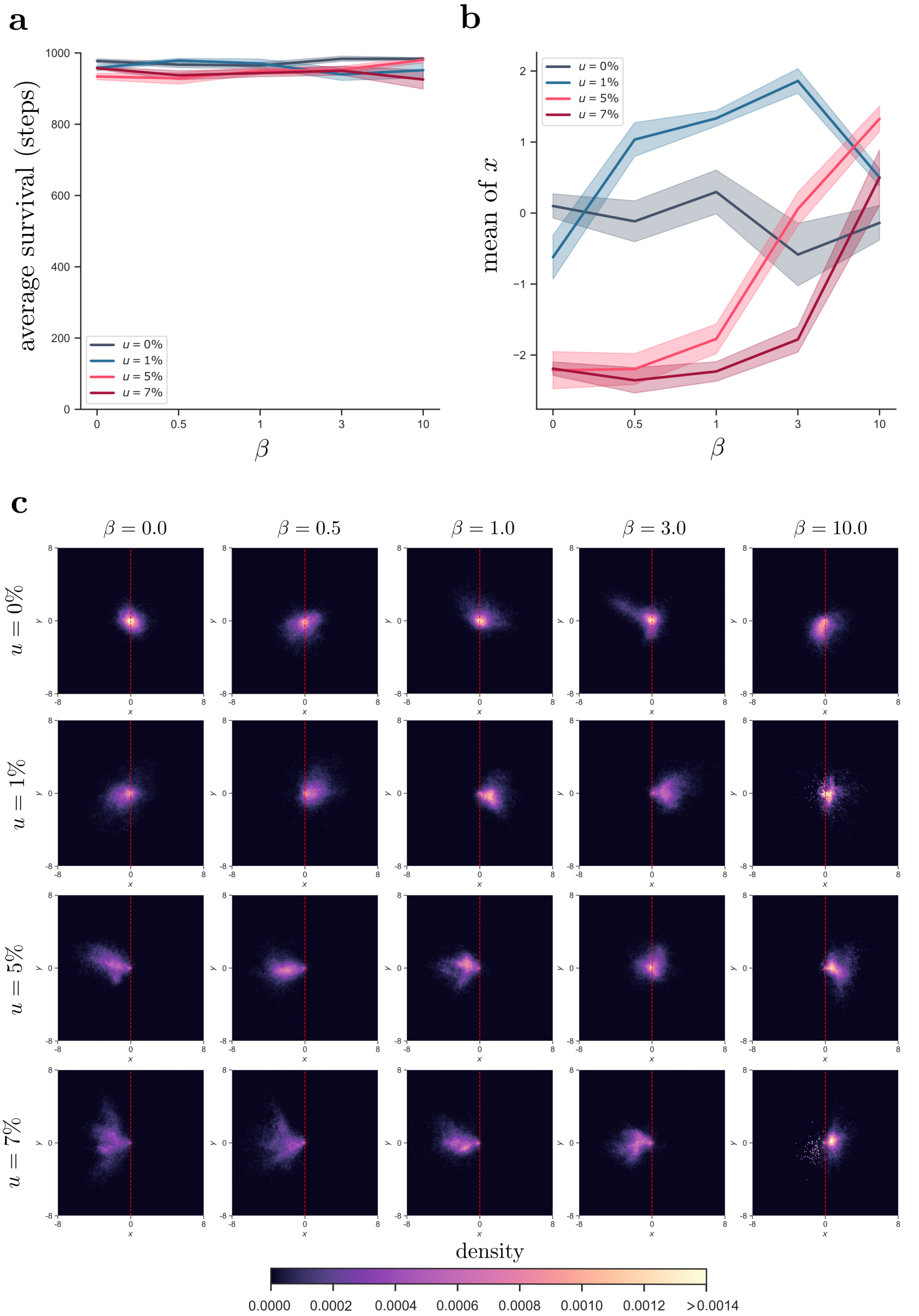

Figure E.5: Ant shows flexible preferences for stochastic transitions in the half plane $x > 0$ as a function of the β parameter, which controls the preference for state transition entropy, for a constant $\alpha = 1$. Averages are across 1000 episodes for each of the 5 different random seeds. (a) Average survival times for the agents show that all agents learned to approximately survive 1000 step episodes. (b) Mean of x position of the ant, as a function of next-state entropy weight β, for various noise magnitudes u. (c) Position heatmaps of all agents for each combination of parameters.

(Fig. E.5b, increasing curves), without much effect on survival rates (Fig. E.5a).

F Differences with KL regularization

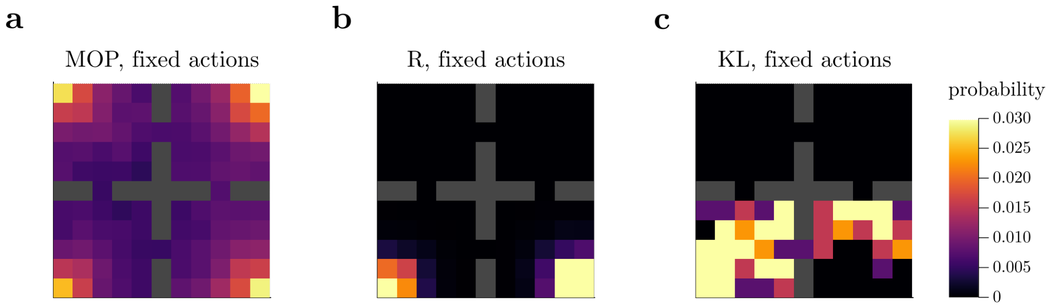

Figure F.6: Fixing the number of actions for non-absorbing states in the gridworld environment, instead of having it be variable across states. We fix this number of actions at 9 for non-absorbing states. (a) MOP agent with fixed actions, (b) R agent with fixed actions, (c) KL regularization agent with fixed actions.

Given the similarity of our objective, Eq. (1) to a KL regularization scheme [79], here we contrast predictions of using a KL divergence (relative entropy) compared to an absolute entropy objective. A relative entropy objective would look like a maximization of the cumulative immediate reward given by the negative KL divergence between a behavioral policy $\pi(a|s)$ and a default policy $\pi_0(a|s)$,

$$-D_{\mathrm{KL}}(\pi(a|s)||\pi_0(a|s)) = \sum_a \pi(a|s) \ln \frac{\pi_0(a|s)}{\pi(a|s)} \tag{F.1}$$

$$= H(\pi(a|s)) - \ln(|\mathcal{A}(s)|), \tag{F.2}$$

where the second equation comes from considering a default policy that is a uniform over actions, conveying the idea that we want to be as close to a uniform policy as possible. However, the lack of an extrinsic reward makes this case degenerate, in the sense that all states are equally preferred. We can see this by realizing that the highest possible immediate intrinsic reward in this case is zero, given that KL divergence is always non-negative. Thus, the optimal policy at all states is uniform over all available actions in each state. For instance, for absorbing states, where only one action is available, Eq. (F.2) is zero, making a “KL agent" be equally attracted to non-absorbing and absorbing states, completely opposite to the motivation of our work, and illustrated in Supplemental Fig. F.6c, where the agent dies very quickly. Furthermore, having a variable or a fixed number of actions for non-absorbing states is the same for a KL agent, since the relative entropy regularizes over the number of actions. This is in stark contrast with MOP, which intrinsically prefers states with a high number of actions. Having a fixed number of actions for non-absorbing states affects the behavior of MOP agents, which we can see in our gridworld experiment, comparing Fig. 2b and Fig. F.6.

Finally, one could imagine setting up a default policy for a KL agent with a different set of available actions than the behavioral policy. In particular, we can set the action set for the default uniform policy to be fixed everywhere, including absorbing states. This amounts to shifting the immediate reward by a scalar everywhere, resulting in an equivalent objective as MOP However, it is hard to see how one can justify allowing the default policy to have a different set of actions than the behavioral policy, especially because of the sum over actions in Eq. (F.2) implies that we sum over all (im)possible actions and implicitly set the probability $\pi(a|s)$ of the behavioral policy to be zero for actions that are not in its support. In contrast, MOP does not have to deal with this problem, and can easily handle constant or variable number of actions for non-absorbing states.

G Comparison to Empowerment and Active Inference

G.1 Empowerment

In this subsection we compare the behaviors attained by the MOP and empowered (MPOW) agents. We implemented empowerment for the 4-room gridworld and cartpole experiments.

G.1.1 4-room gridworld

For the 4-room gridworld, we implemented empowerment in its original discrete formulation [20]. That is, we take the definition of empowerment of a particular state s_t at time t as the channel capacity between the agent's n-step actions $a_t^n = (a_t, a_{t+1}, ..., a_{t+n-1}) \in \mathcal{A}^n$ at this state, and the resulting states s_{t+n},

$$\mathcal{C}(s_t) = \max_{p(a_t^n|s_t)} \sum_{\mathcal{A}^n, \mathcal{S}} p(s_{t+n}|s_t, a_t^n) p(a_t^n|s_t) \log \left(\frac{p(s_{t+n}|s_t, a_t^n)}{\sum_{\mathcal{A}^n} p(s_{t+n}|s_t, a_t^n) p(a_t^n|s_t)} \right), \tag{G.1}$$

where $p(a_t^n|s_t)$ is the probability distribution of n-step actions that mutual information is maximized over, and $p(s_{t+n}|s_t, a_t^n)$ is the n-step world model, computed as

$$p(s_{t+n}|s_t, a_t^n) = p(a_t^n|s_t) \prod_{\tau=0}^{n-1} p(s_{t+\tau+1}|s_{t+\tau}, a_{t+\tau})$$

.

This maximization procedure is done via the Blahut-Arimoto algorithm [86], with a tolerance of 1×10^{-12} for $\|p_{k+1}(a_t^n|s_t) - p_k(a_t^n|s_t)\|$, where k is the iteration number of the algorithm. The initial condition for the n-step action probabilities is uniform over actions, and for this particular environment, very few iterations were needed for convergence (typically 3 or 4).

We initialize an agent at a particular location (in the center of a room, $(x, y) = (3, 3)$), with an internal energy of $E = 30$, so that the initial state is $s = (E, x, y) = (30, 3, 3)$. The agent looks ahead at all possible immediately successor states s_{t+1}, computes their empowerment, and greedily chooses the action that corresponds to the successor state with highest empowerment (environment is deterministic). In our particular formulation, we allowed for a stochastic choice of action in case of empowerment ties between successor states. Note that the behavioral policy (greedy maximization of empowerment) and the probability of the n-step actions over which mutual information is maximized are different [20].

Given the nature of the arena, we implemented 5-step empowerment, to give the agent enough lookahead to consider going into other rooms, while keeping the computations tractable, given the large amount of 1-step actions (9 for center cells). Usually, empowerment assumes a fixed amount of actions across states, and simply considers inconsequential actions to end in the current state, such as running into a wall resulting in staying in the same place. We implemented this original formulation of empowerment, although it is possible to implement state-dependent action sets, as for our formulation of MOP. This would still be meaningful for empowerment, as having more actions available results in more distinct successor states, producing similar predictions as in the original formulation of empowerment.

G.1.2 Cartpole

For the case of the cartpole experiment, we implemented continuous-state empowerment, as developed in [73],

$$\mathcal{C}(s_t) = \max_{p(a_t^n|s_t)} \sum_{\mathcal{A}^n} p(a_t^n|s_t) \int_{\mathcal{S}} p(s_{t+n}|s_t, a_t^n) \log \left(\frac{p(s_{t+n}|s_t, a_t^n)}{\sum_{\mathcal{A}^n} p(s_{t+n}|s_t, a_t^n) p(a_t^n|s_t)} \right) \mathrm{d}s_{t+n}, \tag{G.2}$$

where $p(s_{t+n}|s_t, a_t^n)$ is now a probability density over successor states s_{t+n}.

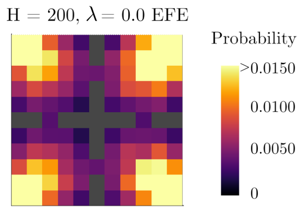

Figure G.7: In our grid-world environment, the expected free energy (EFE) agent only visits a restricted portion of the arena, as long as the target distribution is not perfectly uniform (see Supplemental Sec. G.2.2). In the limit of infinite temperature ($\lambda = 0$), the EFE degenerates to a survival maximization and the stochasticity is due to the Free Energy degeneracy across actions.

In order to have enough lookahead without needing high n, we used 3-step empowerment with each action in the 3-step action held constant for $k = 10$ time steps, in order for the computation of empowerment to be meaningfully different between states. Following [73], we constructed a Gaussian process from where successor states s_{t+n} can be drawn for each of the actions, only in the computation of empowerment (real dynamics are still deterministic). The standard deviation of the noise that blurs successor states was $\sigma = 0.01\mathbf{I}_{4\times 4}$, as in [73], independent of the action. The number of Monte Carlo samples needed to be drawn to approximate the high dimensional integral in Eq. (G.2) was $N_{MC} = 300$. The computation of empowerment is done similarly as in the gridworld, through a Blahut-Arimoto algorithm described in [73]. Similarly, the agent looks ahead at successor states, computes their empowerment and greedily chooses the action that corresponds to the state with the highest empowerment.

G.2 Active Inference

Second, we compared with an active inference approach [88]. Note that our experiments assume full observability of states, although the partial observability condition has often been studied under active inference [89]. The Expected Free Energy (EFE) is defined as the quantity

$$G_{\pi,t}(s_t) = \sum_{\bar{s}_{t+1},\bar{a}_t} p_\pi(\bar{s}_{t+1}, \bar{a}_t|s_t) \log \frac{p(\bar{s}_{t+1}|\bar{a}_t, s_t)}{q(\bar{s}_{t+1})}, \tag{G.3}$$

which is to be minimized as a function of the policy π, which is allowed to change as a function of the state. Here $\bar{s}_{t+1} = (s_{t+1}, s_{t+2}, ..., s_T)$ and $\bar{a}_t = (a_t, a_{t+1}, ..., a_{T-1})$, that is, the sequence of future states and actions respectively from time t up to some finite time T given that the initial state at time t is s_t. Thus, $p_\pi(\bar{s}_{t+1}, \bar{a}_t|s_t)$ and $p(\bar{s}_{t+1}|\bar{a}_t, s_t)$ refer to the join probability of future states and actions, and their conditional, respectively, given the initial state. The quantity $q(\bar{s}_{t+1})$ factorizes as $q(\bar{s}_{t+1}) = \prod_{\tau=t}^{T-1} q(s_{\tau+1})$, where $q(s)$ is a time-independent probability describing the "desired" states of the agent, capturing the idea that desired states are independent of time. Note that $G_{\pi,t}(s_t)$ is the expectation over actions given a policy π of the KL divergence between $p(\bar{s}_{t+1}|\bar{a}_t, s_t)$ and $q(\bar{s}_{t+1})$, that is, $G_{\pi,t}(s_t) = \mathbb{E}_{\bar{a}_t\sim\pi} KL(p(\bar{s}_{t+1}|\bar{a}_t, s_t)||q(\bar{s}_{t+1}))$. Note that because the time horizon is finite, here we need to consider time-dependent policies, so $\pi(a_t|s_t)$ is understood as the probability of selecting action a_t at time t given that the state at time t is s_t. Time-independent policies will be suboptimal in general in finite horizon MDPs.

Minimizing the objective in Eq. (G.3) is similar to MOP in that state transition entropy is being maximized, but it differs in that there is no action entropy and there is a regularizing distribution $q(s)$ towards which states should converge on the long run. The latter distinction highlights a difference in focus of the EFE and MOP approaches, but they can be made similar by just taking $q(s)$ to be uniform in state space. However, the former difference is essential: the optimal policy of the EFE will be deterministic (see Sec. G.2), while the optimal policy of MOP is stochastic. Therefore, one expects to find much larger behavioral variability under MOP than under EFE with uniform preference over all states.

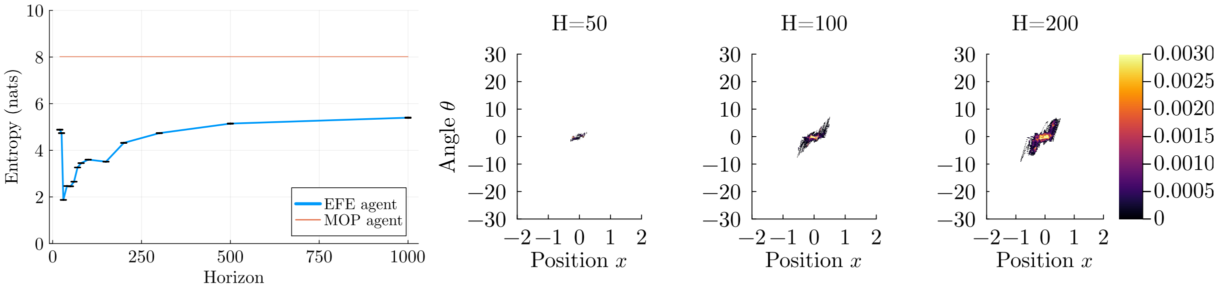

Figure G.8: (Left) Entropy of distribution of visited state space as a function of horizon shows EFE agent is never as good as the MOP agent in generating variability. (Right) Increasing the horizon lookahead makes EFE agent similar to the R agent described in the main manuscript (see Fig. 4)

By virtue of the Markov property, we have $p_\pi(\bar{s}_{t+1}, \bar{a}_t|s_t) = \prod_{\tau=t}^{T-1} \pi(a_\tau|s_\tau)p(s_{\tau+1}|s_\tau, a_\tau)$ and $p(\bar{s}_t|\bar{a}_t, s_t) = \prod_{\tau=t}^{T-1} p(s_{\tau+1}|s_\tau, a_\tau)$. Therefore, the objective in Eq. (G.3) can be recursively written as

$$G_{\pi,t}(s_t) = \sum_{s_{t+1},a_t} \pi(a_t|s_t)p(s_{t+1}|s_t, a_t) \left[\log \frac{p(s_{t+1}|s_t, a_t)}{q(s_{t+1})} + G_{\pi,t+1}(s_{t+1})\right] \tag{G.4}$$

for $t < T - 1$, while the terminal value is

$$G_{\pi,T-1}(s_{T-1}) = \sum_{s_T,a_{T-1}} \pi(a_{T-1}|s_{T-1})p(s_T|s_{T-1}, a_{T-1}) \log \frac{p(s_T|s_{T-1}, a_{T-1})}{q(s_T)}, \tag{G.5}$$

as at time T the episode terminates.

Note that the above formalization slightly generalizes EFE [88] by allowing the possibility that the optimal policy is stochastic. Next we show that the optimal policy is deterministic.

To find the optimal policy, we proceed backwards in time [14]. At time $T - 1$ the optimal policy is deterministic because Eq. (G.5) is linear in the policy. The only exception is that there could be ties between several actions having the same value of the objective, in which case one can be always chosen arbitrarily, or they can be chosen randomly. Therefore, the optimal action is

$$a^*_{T-1}(s_{T-1}) = \arg\min_a \sum_{s_T} p(s_T|s_{T-1}, a) \log \frac{p(s_T|s_{T-1}, a)}{q(s_T)} \tag{G.6}$$

and define the optimal return at time $T - 1$ as

$$G^*_{T-1}(s_{T-1}) = \sum_{s_T} p(s_T|s_{T-1}, a^*_{T-1}(s_{T-1})) \log \frac{p(s_T|s_{T-1}, a^*_{T-1}(s_{T-1}))}{q(s_T)}, \tag{G.7}$$

Proceeding backwards, with $t = T - 2, T - 3, ...$, we find that again for all times the optimal policy is deterministic, and that the optimal action is

$$a^*_t(s_t) = \arg\min_a \sum_{s_{t+1}} p(s_{t+1}|s_t, a) \left[\log \frac{p(s_{t+1}|s_t, a)}{q(s_{t+1})} + G^*_{t+1}(s_{t+1})\right], \tag{G.8}$$

where the optimal return is recursively computed as

$$G^*_t(s_t) = \sum_{s_{t+1}} p(s_{t+1}|s_t, a^*_t(s_t)) \left[\log \frac{p(s_{t+1}|s_t, a^*_t(s_t))}{q(s_{t+1})} + G^*_{t+1}(s_{t+1})\right]. \tag{G.9}$$

G.2.1 Discounted infinite-horizon sophisticated inference is identical to reward maximization under deterministic dynamics

Here, we show that under an infinite horizon, a discounted expected free energy that considers state-dependent policies in the future is equivalent to reward maximization under deterministic dynamics. We start with the same assumption as before that minimizing EFE optimally needs to consider future states where the agent is minimizing EFE. In a discounted, infinite horizon case, this becomes

$$G_\pi(s) = \sum_{s',a} \pi(a|s) p(s'|s,a) \left[\log \frac{p(s'|s,a)}{q(s')} + \gamma G_\pi(s')\right], \tag{G.10}$$

where $\gamma < 1$. Under deterministic dynamics $p(s'|s,a) = 1$ for only one state s', i.e. $s' = s'(s,a)$. So we can rewrite the EFE as

$$G_\pi(s) = \sum_{a} \pi(a|s) \left[-\log\left(q(s'(s,a))\right) + \gamma G_\pi(s'(s,a))\right]. \tag{G.11}$$

Asking to minimize G is equivalent to maximizing $-G$, which means that the optimal Bellman equation for sophisticated active inference in this case turns to

$$G^*(s) = \max_a \left[\log\left(q(s'(s,a))\right) + \gamma G^*(s'(s,a))\right]. \tag{G.12}$$

Simply rewriting $\log(q(s'(s,a))) = r(s'(s,a))$ gives us the typical Belllman equation for MDPs.

In particular, when the preferred distribution is uniform on a finite portion of state space, under the presence of absorbing states outside this portion, this scheme is identical to survival maximization. This is because we can define $q(s'(s,a)) = 1/V$, where V is the volume of the portion of state space that is not absorbing, for $s'(s,a)$ that stays in this portion. For states outside this region, we can establish $q(s'(\text{absorbing})) \ll q(s'(\text{alive}))$, such that $\log(q)$ is bounded. Therefore, for long horizons, we expect the EFE agent to behave identically to our previously defined R agent that maximizes survival. We confirm this expectation in Supplemental Fig. G.8.

G.2.2 Details of simulations

One can define a target distribution $q(s)$ through a Boltzmann distribution, instead of a hard maximization of rewards, as similar to what is done in soft RL [48, 49]. The target distribution $q(s)$ can be defined as

$$q_\lambda(s) = \frac{1}{Z_\lambda} \exp(\lambda R(s)), \tag{G.13}$$

where λ is an inverse temperature, which expresses how motivated the agent is to maximize reward [88].

Grid world We take $R = \delta$ for being in the food and $R = 0$ otherwise. For a large temperature, λ is small, and thus $q_\lambda(s)$ is very close to an uniform distribution –it has a little bump on the reward location. Even a tiny bump breaks the symmetry of the EFE agent in deterministic environments such that it absolutely prefers the food source location, and thus behavior collapses to the occupancy of that single state (see Fig. 6).

Cartpole We define the rewards similar to the R agent, $R = 1$ for non-absorbing states and $R = 0$ for absorbing states. This amounts to a uniform target distribution $q(s)$ over non-absorbing states.

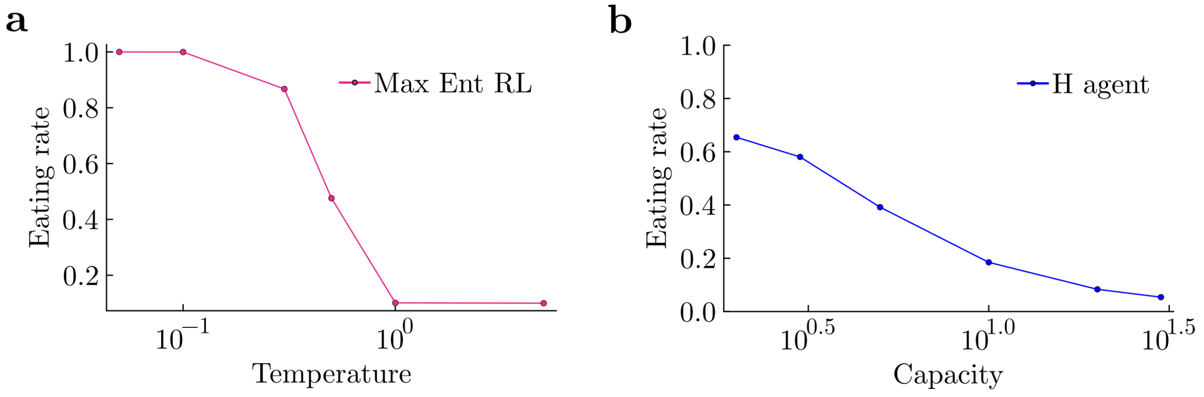

Figure H.9: Reward is not necessary for "goal-directed" behavior. (a) Eating rate as a function of the temperature parameter α in Equation (H.1) for a MaxEnt RL agent in the four-room grid world. (b) Eating rate as a function of the capacity for a MOP agent in the four-room grid world.

H Relationship to Maximum Entropy Reinforcement Learning and goal directedness

The objective of maximizing action-state path entropy in Eq. (2) for the special case $\beta = 0$ can be obtained from the maximum entropy reinforcement learning (MaxEnt RL) formulation [46, 47, 49]

$$V_\pi(s) = \mathbb{E}_\pi \left[\sum_{t=0}^{\infty} \gamma^t \left(r(s_t, a_t) + \alpha \mathcal{H}(\pi(\cdot|s_t)) \right) \Big| s_0 = s \right], \tag{H.1}$$

by setting the reward $r(s,a) = 0$ for all states and actions, and therefore there is no difference between the two approaches in this particular case. However, this reduction obscures the fact that we can generate goal-directed behaviors in H-agents *without* the need of specifying rewards –indeed, this is one of the main accomplishment of our work. To see this, we first quantify how a MaxEnt RL agent gets reward in the four-room grid world defined in Supplemental Sec. E.3, as a function of the temperature parameter α. In this case, a sensible goal is “eating food” (that is, defining $r(s,a) = 1$ at the food locations, and zero everywhere else). Trivially, when $\alpha \ll 1$ in Eq. (H.1), the goal is simply to maximize the future expected reward, equivalent to the ϵ-greedy R agent defined in Supplemental Sec. E.2, for $\epsilon = 0$ (Figure H.9a, leftmost points). In contrast, for $\alpha \gg 1$, we recover the MOP agent in practice (due to the environment being deterministic). In this case, the agent mostly focuses on maximizing future expected entropy, and getting small eating rate (Figure H.9a, rightmost points). Therefore, the temperature α quantifies how “goal directed” the agent should be, where the goal here is understood as getting food, and the entropy term is understood as a regularizer that promotes exploration of the arena.

To aid in showing our central result that an extrinsic reward is not necessary for “goal directed behavior”, we take the MOP agent and vary its energy capacity (see Supplemental Sec. E.3). For large capacities, the MOP agent can largely ignore the food most of the time, obtaining small eating rate (Figure H.9b, right-most points). This is because food is conceived as the means to accomplish the goal of maximizing future path occupancy. In contrast, when capacity is small, the MOP agent needs to get the food much more frequently to avoid the absorbing state, thus getting much higher eating rates (Figure H.9b, leftmost points). The remarkably strong qualitative similarities between the two panels in the figure show that by reinterpreting the concept of reward, one can forego the need of specifying a reward function, and focus on more universal principles of behavior.

I Non-additivity of mutual information and channel capacity

Here we show that mutual information over Markov chains does not obey the additive property. It suffices to prove our statement for paths of length two. Thus, we ask whether the mutual information between actions (a_0, a_1) and states (s_1, s_2) given initial state s_0

$$\mathrm{MI}_{\text{global}} = \sum_{a_0,a_1,s_1,s_2} p(a_0, s_1, a_1, s_2|s_0) \ln \frac{p(a_0, s_1, a_1, s_2|s_0)}{p(a_0, a_1|s_0)p(s_1, s_2|s_0)}$$

equals the sum of the per-step mutual information

$$\mathrm{MI}_{\text{local}} = \sum_{a_0,s_1} p(a_0, s_1|s_0) \ln \frac{p(a_0, s_1|s_0)}{p(a_0|s_0)p(s_1|s_0)} + \sum_{a_0,a_1,s_1,s_2} p(a_0, s_1, a_1, s_2|s_0) \ln \frac{p(a_1, s_2|s_1)}{p(a_1|s_1)p(s_2|s_1)}$$

where $p(a_0, s_1, a_1, s_2|s_0) = \pi(a_0|s_0)p(s_1|s_0, a_0)\pi(a_1|s_1)p(s_2|s_1, a_1)$ and $p(a_0, s_1|s_0) = \pi(a_0|s_0)p(s_1|s_0, a_0)$. Using Bayes' rule and the Markov property, the above quantities can be rewritten as

$$\begin{aligned}
\mathrm{MI}_{\text{global}} &= \sum_{a_0,a_1,s_1,s_2} p(a_0, s_1, a_1, s_2|s_0) \ln \frac{p(a_0, a_1|s_0, s_1, s_2)}{p(a_0, a_1|s_0)} \\
&= \sum_{a_0,a_1,s_1,s_2} p(a_0, s_1, a_1, s_2|s_0) \ln \frac{p(a_0|s_0, s_1)p(a_1|s_1, s_2)}{p(a_0, a_1|s_0)} \\
&= \sum_{a_0,a_1,s_1,s_2} p(a_0, s_1, a_1, s_2|s_0) \ln \frac{p(a_0|s_0, s_1)p(a_1|s_1, s_2)}{\pi(a_0|s_0)p(a_1|s_0, a_0)} \\
&= \sum_{a_0,a_1,s_1,s_2} p(a_0, s_1, a_1, s_2|s_0) \ln \frac{p(a_0|s_0, s_1)p(a_1|s_1, s_2)}{\pi(a_0|s_0)\sum_s \pi(a_1|s)p(s|s_0, a_0)} \\
&= \sum_{a_0,s_1} p(a_0, s_1|s_0) \ln \frac{p(a_0|s_0, s_1)}{\pi(a_0|s_0)} + \sum_{a_0,a_1,s_1,s_2} p(a_0, s_1, a_1, s_2|s_0) \ln \frac{p(a_1|s_1, s_2)}{\sum_s \pi(a_1|s)p(s|s_0, a_0)}
\end{aligned}$$

and

$$\mathrm{MI}_{\text{local}} = \sum_{a_0,s_1} p(a_0, s_1|s_0) \ln \frac{p(a_0|s_0, s_1)}{\pi(a_0|s_0)} + \sum_{a_0,a_1,s_1,s_2} p(a_0, s_1, a_1, s_2|s_0) \ln \frac{p(a_1|s_1, s_2)}{\pi(a_1|s_1)}$$

The quantities $\mathrm{MI}_{\text{global}}$ and $\mathrm{MI}_{\text{local}}$ are remarkable similar except for the denominator in the $\ln$ of the last term in each expression. Therefore, equality between $\mathrm{MI}_{\text{global}}$ and $\mathrm{MI}_{\text{local}}$ holds iff

$$\sum_{a_0,a_1,s_1,s_2} p(a_0, s_1, a_1, s_2|s_0) \ln \sum_s \pi(a_1|s)p(s|s_0, a_0) = \sum_{a_0,a_1,s_1,s_2} p(a_0, s_1, a_1, s_2|s_0) \ln \pi(a_1|s_1),$$

which is not true for all choices of policy and transitions probabilities. To see this, take a Markov chain where the action $a_0 = 0$ from $s_0 = 0$ is deterministic, but results in two possible successor states $s_1 = 1$ or $s_1 = 2$ with equal probability $1/2$. From $s_1 = 1$ the policy takes actions $a_1 = 1$ and $a_1 = 2$ with probability $1/2$. From $s_1 = 2$ the policy is deterministic, that is, $a_1 = 3$ with probability 1. A simple calculation shows that the left side equals $-\frac{3}{2} \ln 2$, while the right side equals a different quantity, $-\frac{1}{2} \ln 2$.
