# Supplementary material for: Complex behavior from intrinsic motivation to occupy future action-state path space
Source: Nat Commun. 2024 Jul 29;15:6368. doi: 10.1038/s41467-024-49711-1 (PMC11286966; doi:10.1038/s41467-024-49711-1)
Supplement: Supplementary file 3 — Description Of Additional Supplementary File [file 41467_2024_49711_MOESM3_ESM.pdf]

Description of Additional supplementary file

Supplementary movie 1. Animation of a portion of an episode comparing the behaviors of the MOP agent and the ϵ-greedy R agent for the four-room grid world environment (see main text, Fig. 2 for more details).

Supplementary movie 2. Animation of a portion of an episode of the MOP agent (mouse) behaving in the predator-prey scenario detailed in Fig. 3.

Supplementary movie 3. Animation of a portion of an episode of the R agent (mouse) behaving in the predator-prey scenario detailed in Fig. 3.

Supplementary movie 4. Animation of a portion of an episode comparing the behaviors of the MOP agent and the R agent for the cartpole experiment detailed in Fig. 4.

Supplementary movie 5. Animation of the state space trajectories (in an angle-position projection) traveled by the MOP and the R agents from Video 4, sped up four times the original frame rate.

Supplementary movie 6. Animation of a portion of an episode comparing the behaviors of the MOP agent and the lifetime-matching $\epsilon- $greedy R agent for the cartpole experiment detailed in Fig. 4.

Supplementary movie 7. Animation of a portion of an episode comparing the behaviors of the MOP agent and the MPOW and EFE agents for the four-room grid world environment (see main text, Fig. 6 for more details).

Supplementary movie 8. Animation of a portion of an episode comparing the behaviors of the MOP agent and the MPOW and EFE agents for the cartpole experiment (corresponding to Fig. 6).

Supplementary movie 9. Animation of a portion of an episode comparing the behaviors of the MOP agent and the R agent for the quadruped experiment without energetic constraints (corresponding to upper row of Fig. 7).

Supplementary movie 10. Animation of a portion of an episode comparing the behaviors of the MOP agent and the R agent for the quadruped experiment with energetic constraints (corresponding to lower row of Fig. 8).
